# Complete characterization of ultrafast optical fields by phase-preserving nonlinear autocorrelation

**DOI:** 10.1038/s41377-022-00978-3

**Published:** 2022-09-20

**Authors:** Alexander Gliserin, Soo Hoon Chew, Seungchul Kim, Dong Eon Kim

**Affiliations:** 1 Department of Optics and Mechatronics Engineering, College of Nanoscience and Nanotechnology, Pusan National University, 2 Busandaehak-ro 63beon-gil, Busan, 46241 South Korea; 2 Department of Cogno Mechatronics Engineering, College of Nanoscience and Nanotechnology, Pusan National University, 2 Busandaehak-ro 63beon-gil, Busan, 46241 South Korea; 3 Max Planck Center for Attosecond Science, Max Planck POSTECH/Korea Research Initiative, 77 Cheongam-ro, Pohang, 37673 South Korea; 4 Department of Physics, Center for Attosecond Science and Technology, Pohang University of Science and Technology, 77 Cheongam-ro, Pohang, 37673 South Korea

**Keywords:** Nonlinear optics, Ultrafast photonics, Optical metrology

## Abstract

Nonlinear autocorrelation was one of the earliest and simplest tools for obtaining partial temporal information about an ultrashort optical pulse by gating it with itself. However, since the spectral phase is lost in a conventional autocorrelation measurement, it is insufficient for a full characterization of an ultrafast electric field, requiring additional spectral information for phase retrieval. Here, we show that introducing an intensity asymmetry into a conventional nonlinear interferometric autocorrelation preserves some spectral phase information within the autocorrelation signal, which enables the full reconstruction of the original electric field, including the direction of time, using only a spectrally integrating detector. We call this technique *Phase-Enabled Nonlinear Gating with Unbalanced Intensity* (PENGUIN). It can be applied to almost any existing nonlinear interferometric autocorrelator, making it capable of complete optical field characterization and thus providing an inexpensive and less complex alternative to methods relying on spectral measurements, such as frequency-resolved optical gating (FROG) or spectral phase interferometry for direct electric-field reconstruction (SPIDER). More importantly, PENGUIN allows the precise characterization of ultrafast fields in non-radiative (e.g., plasmonic) nonlinear optical interactions where spectral information is inaccessible. We demonstrate this novel technique through simulations and experimentally by measuring the electric field of ~6-fs laser pulses from a Ti:sapphire oscillator. The results are validated by comparison with the well-established FROG method.

## Abstract (web summary)

We introduce and experimentally demonstrate a novel technique for the complete characterization of ultrafast optical fields based on phase-preserving nonlinear interferometric autocorrelation without any spectroscopic measurements.

## Graphical abstract

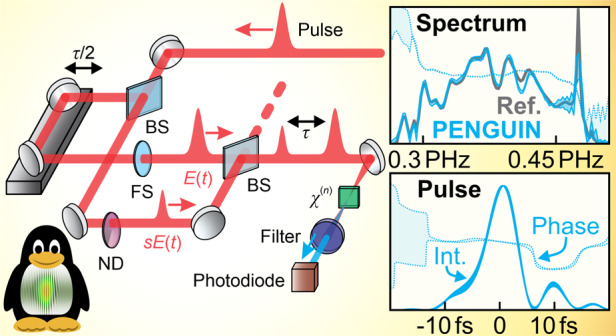

## Introduction

The rapid advancement of ultrafast laser sources over the past decades^1,2^ has enabled the direct observation and control of the fastest light-matter interactions on their natural time scales leading to a wide variety of scientific and industrial applications^3,4^. Lasers producing few- and single-cycle optical pulses are readily available^5–9^, and even sub-cycle optical transients have been achieved using well-controlled synthesized electric fields^10,11^. The precise characterization of these ultrafast and broadband electric fields is crucial for most applications in science and technology; yet, a direct measurement of the shortest optical pulses is technically challenging, since it requires an even shorter sampling process, such as attosecond streaking with extreme ultraviolet pulses^2,12–15^ or tunneling ionization^16^, which is limited to strong optical fields.

Historically, short optical pulses were first measured indirectly via nonlinear autocorrelation^17–21^, yielding only rough pulse shape parameters. The fringe-resolved or interferometric autocorrelation (IAC) variant, first reported by Diels et al.^22,23^, contains some information about the chirp^24–26^ and enabled for the first time the full reconstruction of the original optical field in many practical cases^5,27–30^ except for the direction of time. Unbalanced autocorrelation, although considered detrimental at first^31^, has been shown to mitigate this ambiguity, either by modulating the phase^32^ or the amplitude^33^ in one of the interferometer’s arms. However, none of these methods preserve (or make use of) any spectral phase information in the autocorrelation signal itself, and therefore require the independently measured fundamental spectrum of the pulse for phase retrieval.

Frequency-resolved optical gating (FROG)^34,35^ enables the full retrieval of an arbitrary optical field from a single measurement by combining a nonlinear autocorrelation with spectroscopy. This technique measures the optical spectrum of the nonlinear autocorrelation signal for every delay step, which provides a two-dimensional spectrogram. Although the spectral phase is lost in the FROG spectrogram just as in the case of a spectrally integrated nonlinear autocorrelation, a unique solution for the electric field is usually guaranteed in a two-dimensional phase retrieval problem except for trivial ambiguities such as the constant carrier-envelope phase (CEP) or the direction of time in certain configurations^35^. The more recent interferometric FROG variants^36,37^, i.e., the spectrally resolved extension of the IAC, are suitable for the shortest optical pulses, since they eliminate the geometric broadening present in a conventional non-collinear FROG arrangement; however, they require high sampling accuracy and a more complex field retrieval process^37–39^. Other notable characterization techniques include streaking-type time lens methods for direct temporal measurements, albeit limited to picosecond pulses^40^, dispersion scanning (d-scan)^41,42^, and the widely used spectral phase interferometry for direct electric-field reconstruction (SPIDER)^43,44^. While d-scan yields a spectrogram similar to FROG and requires an iterative retrieval algorithm, SPIDER allows a direct spectral phase measurement but has a significantly more complex optical setup for sum-frequency generation between the pulse itself and a quasi-monochromatic reference at two different frequencies.

All the aforementioned methods require spectroscopy of the nonlinear interaction or knowledge of the spectral intensity of the pulse itself, or both. While spectroscopy is a well-established optical technique, the capability of using a spectrally integrating detector is advantageous for applications involving very weak optical signals or requiring a very high dynamic range offered, e.g., by photomultipliers. A large portion of the optical signal is inherently lost at the input slit and dispersive element of a monochromator, and the pixelated sensors typically used in spectrometers offer only a limited sensitivity and signal gain. This is exacerbated for overdetermined characterization techniques based on two-dimensional spectrograms, such as FROG and d-scan, which provide signal redundancy for the field retrieval but require on the order of *N* times more signal collection (*N* being the number of delay points) than for a one-dimensional autocorrelation to achieve a comparable signal-to-noise ratio. Furthermore, spectrally resolved optical detection is only available if the underlying nonlinear interaction produces detectable light. Non-radiative nonlinear interactions of ultrafast electric fields are therefore inaccessible by spectroscopic characterization techniques. For example, optically induced ultrafast plasmonic near-fields at metallic nanostructures produce low-energy photoelectrons via nonlinear photoemission^45–47^, which is an incoherent process and therefore does not preserve sufficient spectral information about the plasmonic near-fields in the kinetic energy spectrum of the photoelectrons for field retrieval with FROG or other spectroscopic methods. A spectrally integrated nonlinear IAC signal of the plasmonic near-fields, on the other hand, is easily obtained by recording the nonlinear photoemission rate as a function of the autocorrelation delay;^47–49^ however, the extraction of the underlying plasmonic electric fields from such a measurement has been impossible because of inaccessible spectral information or required sub-cycle sampling pulses^50,51^.

Here, we propose a novel approach for complete and inexpensive characterization of ultrafast optical electric fields without requiring a spectroscopic measurement, which employs unbalanced-intensity nonlinear IAC with a spectrally integrating detector (e.g., a photodiode). The intensity asymmetry is achieved via a neutral-density (ND) filter in one of the interferometer’s arms, which breaks the time-reversal symmetry of a balanced IAC, thus preserving non-trivial spectral phase information of the optical field. This allows retrieval of the original field from such an unbalanced-intensity IAC signal with a self-consistent iterative algorithm using a Fourier relation. We call this technique *Phase-Enabled Nonlinear Gating with Unbalanced Intensity* (PENGUIN). Unlike previous implementations of the unbalanced IAC^32,33^, PENGUIN utilizes the spectral phase information contained in the IAC signal and therefore does not require the fundamental spectrum for field retrieval. The feasibility and limitations of this novel technique are discussed based on numerical simulations. In addition, we experimentally demonstrate the complete field retrieval of few-cycle laser pulses using PENGUIN and validate the results by comparison with the well-established FROG method.

## Results

### Unbalanced-intensity interferometric autocorrelation

**Fig. 1:**
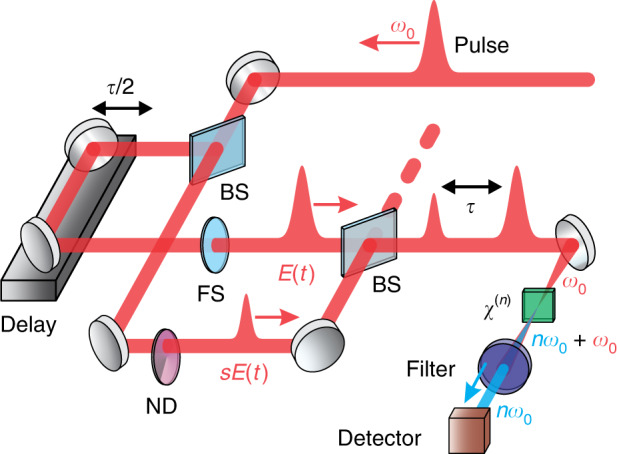
Schematic setup for unbalanced-intensity nonlinear IAC. A collinear Mach–Zehnder interferometer is modified by inserting a variable neutral-density (ND) filter into one arm to adjust its relative field amplitude before *n*th-order harmonic generation. The residual fundamental ($\omega _0$) is removed via a short-pass filter and only the harmonic radiation ($n\omega _0$) is recorded as a function of the delay *τ* by a spectrally integrating detector such as a photodiode. BS beam splitter, FS fused silica plate for dispersion matching, *χ*^(*n*)^: nonlinear medium

Figure 1 shows a typical nonlinear IAC setup based on a dispersion-minimized Mach-Zehnder interferometer, where an optical pulse (fundamental central frequency *ω*_0_) is split into two identical copies with a complex electric field *E*(*t*). The setup is modified by attenuating the pulse in one of the arms via a variable ND filter such that its field amplitude is scaled by a balance factor *s* (*s* ≤ 1). Note that the group delay dispersion (GDD) introduced by the ND filter needs to be matched in the other arm, for example via a fused silica plate. The two pulses interfere with each other since they have identical polarization and are then collinearly focused into an *n*th-order nonlinear medium for parametric *n*th-order harmonic generation (*nω*_0_), e.g., type-I second-order harmonic generation (SHG), which is spectrally separated from the fundamental via short-pass filtering and recorded by a slow spectrally integrating photodetector. The unbalanced-intensity nonlinear (*n*th-order) IAC signal on the detector as a function of the optical delay *τ* is given by

1 $$\begin{array}{l}I_{{{{\mathrm{IAC}}}}}\left( \tau \right) = \displaystyle{\int}_{ - \infty }^{ + \infty } {\left| {\left( {sE\left( t \right) + E\left( {t - \tau } \right)} \right)^n} \right|^2} dt \\\qquad\qquad= \displaystyle{\int}_{ - \infty }^{ + \infty } {\left| {\mathop {\sum }\limits_{k = 0}^n \left( {\begin{array}{*{20}{c}} n \\ k \end{array}} \right)s^kE^k\left( t \right)E^{n - k}\left( {t - \tau } \right)} \right|^2} dt\end{array}$$

The *n*th-order binomial expansion in Eq. (1) results in (*n* + 1)^2^ terms of mixed powers of the delayed and non-delayed electric field and their complex conjugates after the magnitude-squared operation. Therefore, the nonlinear IAC signal is composed of cross-correlations between different powers of the field weighted by the binomial coefficients and powers of *s*. Using the convolution theorem, each cross-correlation integral in the time domain can be expressed as a product of the respective field powers in the frequency domain:

2 $$\begin{array}{l} {I_{{{{\mathrm{IAC}}}}}\left( \omega \right) = \mathop {\sum}\limits_{k = 0}^n {I_{0,k}} \left( \omega \right) + \mathop {\sum}\limits_{m = 1}^n {\mathop {\sum}\limits_{k = 0}^{n - m} {\left[ {I_{m,k}\left( \omega \right) + \overline {I_{m,k}\left( { - \omega } \right)} } \right]} } ,} \\ {I_{m,k}\left( \omega \right) = \left( {\begin{array}{*{20}{c}} n \\ k \end{array}} \right)\left( {\begin{array}{*{20}{c}} n \\ {k + m} \end{array}} \right)s^{2k + m}{{{\mathcal{F}}}}\left\{ {E^m\left( t \right)\left| {E^k\left( t \right)} \right|^2} \right\} }\\\overline {{{{\mathcal{F}}}}\left\{ {E^m\left( t \right)\left| {E^{n - k - m}\left( t \right)} \right|^2} \right\}} \end{array}$$

where ${{{\mathcal{F}}}}$ denotes a Fourier transform from the time domain into the frequency domain, and the top bar denotes complex conjugation. Note that in Eq. (2), the cross-correlation components, $I_{m,k}\left( \omega \right)$, constituting the IAC signal in the frequency domain are grouped by their harmonic order *m* (representing both positive- and negative-frequency orders) from DC (*m* = 0) to the *n*th-order harmonic (*m* = *n*), and the index *k* denotes different components at the same harmonic order. Each of these components directly corresponds to one time-domain integral term, $I_{m,k}\left( \tau \right)$, in Eq. (1). Negative-frequency components for *m* > 0 are reversed replicas of their positive counterparts with a centrosymmetric phase and can therefore be ignored in the subsequent discussion. For a balanced IAC (*s* = 1), it is evident from Eq. (2) that all cross-correlation components at any given harmonic order *m* are either magnitude-squared quantities of the form ${{{\mathcal{F}}}}\left\{ x \right\}\overline {{{{\mathcal{F}}}}\left\{ x \right\}}$ or pairwise complex conjugates of each other, resulting in a purely real IAC spectrum with a flat spectral phase. This loss of spectral phase information is inherent to any type of balanced autocorrelation. In the time domain, this corresponds to time-symmetric or pairwise time-reversed cross-correlation components, respectively, which add up to an overall time-symmetric IAC signal. However, using two pulses with unbalanced intensity (0 < *s* < 1) breaks the symmetry of the pairwise time-reversed cross-correlation components and thus retains spectral phase information within the resulting IAC signal, which enables field retrieval.

**Fig. 2:**
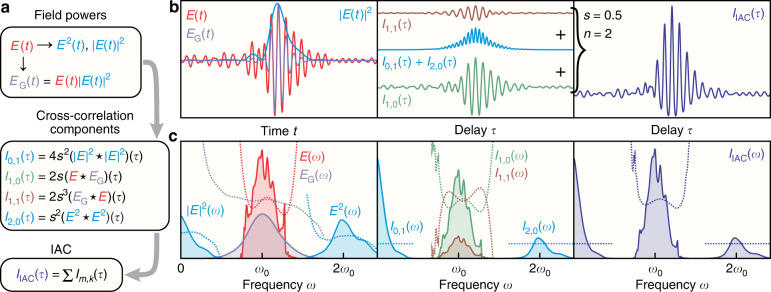
Concept of the unbalanced-intensity nonlinear IAC for *n* = 2. **a** Flow chart of the (mixed) field powers contributing to the different cross-correlation components which constitute the IAC signal in the time domain according to Eq. (1) with harmonic indices as introduced in Eq. (2). The star operator denotes cross-correlation: $\left( {f \star g} \right)\left( \tau \right) = {\int} {\overline {f\left( {t - \tau } \right)} } g\left( t \right)dt$. **b** Time-domain components of the IAC for *n* = 2 and *s* = 0.5 for an exemplary few-cycle pulse (see text) depicting the fundamental and nonlinear field quantities (left panel), the cross-correlation components (middle panel), and the resulting IAC signal (right panel). Only the real parts are shown. **c** Frequency-domain representation of b according to Eq. (2) showing spectral magnitudes (solid lines) and phases (dotted lines)

Let us first consider the lowest nonlinear order, *n* = 2, since it has the fewest cross-correlation components and the simplest experimental implementation. Here, Eqs. (1) and (2) contain only four non-trivial cross-correlation components ($I_{0,1}$, $I_{1,0}$, $I_{1,1}$, and $I_{2,0}$; others are DC constants or at negative frequencies), which are composed of different or mixed field powers, as illustrated in the flow chart in Fig. 2a. An example is shown in Fig. 2b, c in the time and frequency domains, respectively, for *n* = 2 and a balance factor of *s* = 0.5 using the electric field of a typical few-cycle optical pulse (red; left panels) with a realistic spectral magnitude (taken from a measurement) and a synthetic 4th-order polynomial spectral phase close to the Fourier-transform limit (time-bandwidth product of ~1.8). The DC ($I_{0,1}$) and 2nd-order harmonic ($I_{2,0}$) peaks of the IAC contain only one cross-correlation component each (ignoring the delta-like DC constants, $I_{0,0}$ and $I_{0,2}$), resulting in magnitude-squared quantities with flat spectral phases (blue; middle panels). In contrast, the two fundamental ($\omega _0$) cross-correlation components, $I_{1,0}$ and $I_{1,1}$ (green, brown; middle panels), between the fundamental field, $E\left( t \right)$ (red; left panels), and the *gating* field, $E_{{{\mathrm{G}}}}\left( t \right) = E\left( t \right)\left| {E(t)} \right|^2$ in this example (purple; left panels), exhibit spectral phases of opposite sign with respect to each other but similar in shape to the phase of the fundamental field. These two components would be complex conjugates in a balanced IAC (*s* = 1) and thus their phases would cancel out after summation. However, for *s* < 1, their spectral magnitudes differ by a factor of *s*^2^ (or by *s*^2*n*−2^ for any *n*) and a non-trivial spectral phase is retained in the IAC signal (right panel in Fig. 2c). Since knowing one of these two fundamental-frequency components and the balance factor *s* defines the conjugate component, we only consider the component scaled by the lowest power of *s*, $I_{1,0}$, which is given by Eq. (2) for any nonlinear order *n* as

3 $$\begin{array}{l}I_{1,0}\left( \omega \right) = ns{{{\mathcal{F}}}}\left\{ {E\left( t \right)} \right\}\overline {{{{\mathcal{F}}}}\left\{ {E\left( t \right)\left| {E^{n - 1}\left( t \right)} \right|^2} \right\}} \\\qquad\qquad= ns{{{\mathcal{F}}}}\left\{ {E\left( t \right)} \right\}\overline {{{{\mathcal{F}}}}\left\{ {E_{G\left( {1,0} \right)}\left( t \right)} \right\}}\end{array}$$

with $E_{{{{\mathrm{G}}}}\left( {1,0} \right)}\left( t \right) = E\left( t \right)\left| {E^{n - 1}\left( t \right)} \right|^2$ being the *n*th-order gating field for the $I_{1,0}$ component. It is worth noting that this fundamental cross-correlation component contains the most information about the electric field, since it constitutes a cross-correlation between the fundamental field itself and some higher-order gating field, while all other components are cross-correlations between higher powers of the field. It is also intuitive why the spectral shape and phase of the $I_{1,0}$ signal resemble those of the fundamental field for short optical pulses close to the Fourier-transform limit: Since the gating field is significantly shorter in the time domain than the fundamental field (compare the red and purple curves in Fig. 2b, left panel), their cross-correlation (i.e., frequency-domain product) is dominated by the spectral shape and phase of the fundamental field. In fact, for a high enough nonlinear order *n*, the gating field can become delta-like with respect to the fundamental field, resulting in a $I_{1,0}$ signal that accurately reproduces the original field according to Eq. (3). In addition, using a very small balance factor *s* close to zero suppresses all cross-correlation components other than the $I_{1,0}$ signal itself and a constant background ($I_{0,0}$) at the expense of reduced signal contrast. The IAC then directly reproduces the electric field except for a constant offset. Park et al. used this “perturbative” (low *s*) strong-field (high *n*) limit to directly obtain the electric field from a nonlinear unbalanced-intensity IAC measurement without the need for a retrieval algorithm^16^. However, their approach requires very high nonlinear orders *n*, which are only achievable with strong-field effects such as tunneling ionization, and offers only a low dynamic range because of the use of very small balance factors (*s* < 0.03). In contrast, our method works for all *n* ≥ 2 and non-perturbative balance factors close to 1, making it feasible for use with any typical IAC setup.

### Field retrieval

**Fig. 3:**
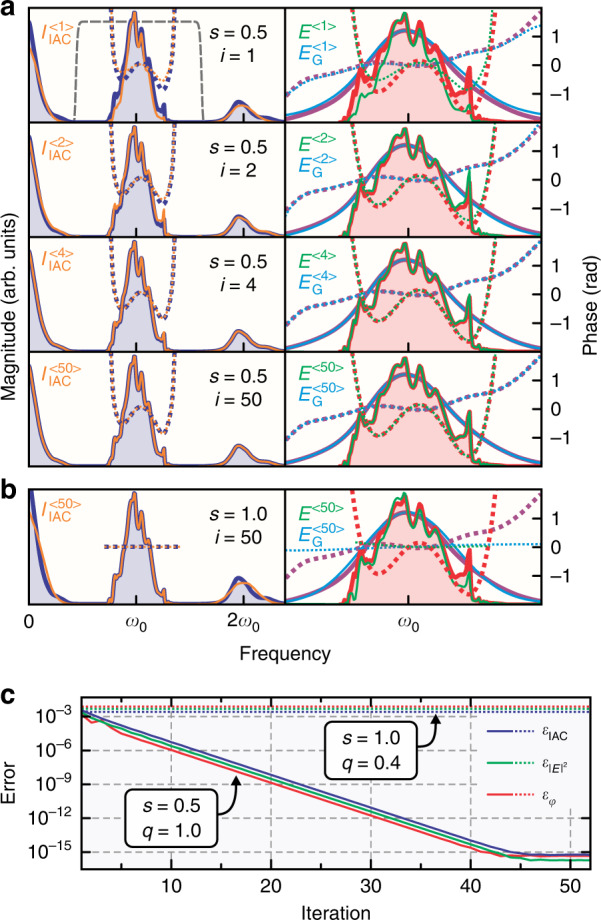
Field retrieval simulation. **a** Field retrieval of a few-cycle low-dispersion pulse from its IAC for *n* = 2 and *s* = 0.5 shown after different iteration steps *i* in the frequency domain. The left column depicts the reference (dark blue) and retrieved (orange) IAC signals as well as the spectral filter (dashed gray line) used to extract the *I*_1,0_ signal and initial field guess from the IAC. The right column shows the reference (red) and retrieved (green) electric fields as well as the reference (purple) and retrieved (blue) gating fields. Magnitudes are shown as solid lines and phases as dotted lines; flat phase segments outside the fundamental peak are not shown. **b** Retrieval attempt from a balanced IAC (*s* = 1) shown after convergence for comparison. Only the fundamental peak of the IAC is reproduced due to the lack of spectral phase information, yielding a wrong field result. **c** Retrieval convergence depicting the error quantities as defined in Eqs. (10–12). The solid lines refer to the *s* = 0.5 case and the dotted lines to the *s* = 1 case. The phase error is shown in radians, while the other errors are dimensionless (but are all shown on the same scale)

In general, the Fourier relation in Eq. (3) between the $I_{1,0}$ signal and the fundamental electric field has no analytic solution. Therefore, the field retrieval is based on iteratively improving an initial field guess to satisfy Eq. (3). Although a variety of numerical optimization algorithms are applicable to this problem, our approach exploits the inherent relationship between the fundamental field and the gating field to optimize the field guess by iteratively evaluating Eq. (3). This avoids the need for a computationally expensive gradient-based numerical optimization of a large parameter set. The choice for the initial field guess is essentially unrestricted (e.g., a Gaussian pulse with a flat phase is possible) as long as it provides sufficient spectral bandwidth to support the retrieved field. Since the fundamental peak of the IAC resembles the spectral shape and phase of the original electric field, it can be directly used as the initial guess, *E*^〈1〉^(*t*), by spectral filtering:

4 $$E^{\left\langle 1 \right\rangle }\left( t \right) = {{{\mathcal{F}}}}^{ - 1}\left\{ {I_{{{{\mathrm{IAC}}}}}\left( \omega \right)F\left( \omega \right)} \right\}$$

with ${{{\mathcal{F}}}}^{ - 1}$ denoting the inverse Fourier transform from the frequency domain into the time domain and *F*(*ω*) being a spectral filter around the fundamental ($\omega _0$) peak (dashed gray line in Fig. 3a). Equation (4) provides a natural choice for the initial guess, since it converges towards $E\left( t \right)$ for large *n* and small *s*. The field guess is updated at the (*i* + 1)th iteration by calculating the gating field using the *i*th-iteration field guess and solving Eq. (3) for the fundamental field:

5 $$E_{{{{\mathrm{G}}}}\left( {1,0} \right)}^{\left\langle i \right\rangle }\left( t \right) = E^{\left\langle i \right\rangle }\left( t \right)\left| {\left[ {E^{\left\langle i \right\rangle }\left( t \right)} \right]^{n - 1}} \right|^2$$

6 $$E^{\left\langle {i + 1} \right\rangle }\left( t \right) = {{{\mathcal{F}}}}^{ - 1}\left\{ {\frac{{I_{1,0}\left( \omega \right)}}{{ns\overline {{{{\mathcal{F}}}}\left\{ {E_{{{{\mathrm{G}}}}\left( {1,0} \right)}^{\left\langle i \right\rangle }\left( t \right)} \right\}} }}} \right\}q + E^{\left\langle i \right\rangle }\left( t \right)\left( {1 - q} \right)$$

where 0 < *q* ≤ 1 is a parameter controlling the rate of convergence that needs to be adjusted to ensure numerical stability depending on the choice of *n* and *s*. The $I_{1,0}$ signal is obtained from the IAC via Eq. (8) either before the retrieval (for *n* = 2) or iteratively during the retrieval (for *n* > 2). (See Materials and Methods.) Essentially, the electric field is decorrelated from the $I_{1,0}$ signal in Eq. (6) in the frequency domain by dividing out the spectrum of the gating field, requiring it to have a non-zero spectral magnitude over the entire fundamental bandwidth, which is true for most practical optical pulses.

Figure 3a depicts the field retrieval at different iteration steps based on Eqs. (4–6) in the frequency domain for a few-cycle pulse using *n* = 2 and *s* = 0.5 (the field in this example is the same as in Fig. 2b, c). A spectral filter (dashed gray line) is used to obtain the initial field guess according to Eq. (4) as well as the $I_{1,0}$ signal (see Materials and Methods) from the reference IAC (dark blue), with a filter width chosen to minimize the retrieval error. The retrieved field (green; right column) rapidly converges towards the reference field (red) within only a few iterations and also yields a perfect match between the reference (purple) and retrieved (blue) gating fields as well as the reference (dark blue; left column) and retrieved (orange) IACs. Since only two Fourier transforms in one dimension are required per iteration step in the simplest case (*n* = 2) according to Eq. (6), the algorithm is computationally fast, and a few iterations are sufficient to reach typical experimental accuracy. For example, one iteration is completed in <1 ms on a typical personal computer for 1000 IAC data points, which allows online diagnostics at video refresh rates. Figure 3b shows the result of the retrieval algorithm applied to a balanced IAC for comparison (*s* = 1, *n* = 2 and the same electric field as in Fig. 3a) after convergence. Here, the $I_{1,0}$ signal cannot be directly extracted from the IAC signal via Eq. (8) before the retrieval; instead, it is iteratively approximated during the retrieval by subtracting the calculated $I_{1,1}$ component (using the current field guess) from the IAC at each iteration. The lack of spectral phase information in the IAC signal leads to an ambiguity in the retrieval, yielding a wrong field solution which satisfies Eq. (3) and thus matches the fundamental spectral peak of the IAC but fails to reproduce its other harmonic peaks. In addition, the convergence parameter in Eq. (6) had to be reduced to *q* = 0.4 to avoid numerical instabilities, while the unbalanced case facilitates the highest rate of convergence (*q* = 1). Figure 3c shows the time-domain IAC errors as well as the intensity and phase errors of the electric field (see Materials and Methods) for each iteration step. The rapid convergence of the unbalanced case (*s* = 0.5, solid lines) continues exponentially over many orders of magnitude until it cuts off around the numerical precision limit of the simulation (~10^−16^), signifying a near-perfect decorrelation of the electric field from the IAC signal. The balanced case (*s* = 1, dotted lines) converges almost immediately but fails to significantly reduce the initial error quantities (~10^−3^), since the wrong field solution is retrieved. Notably, all error quantities show very similar behavior, making the IAC error suitable for assessing the convergence and quality of the field retrieval. This is important for real-world applications of the PENGUIN technique to unknown fields where only the IAC error is accessible.

This simple retrieval method using only the $I_{1,0}$ signal is limited to low-dispersion pulses near the Fourier-transform limit where the gating field is spectrally broader than the fundamental field. For highly dispersed pulses, this algorithm converges to a wrong solution where the retrieved field only reproduces the fundamental peak of the IAC but not its other harmonics, similar to the *s* = 1 case. This dispersion limit depends on both the bandwidth as well as the spectral distribution of the GDD of the pulse; hence, it cannot be universally quantified by a maximum time-bandwidth product for successful retrieval. For example, for the spectral magnitude used in Fig. 3 and a purely linear chirp, i.e., a constant GDD, the maximum acceptable time-bandwidth product is ~1.6 (lower than that of the polynomial phase example in Figs. 2 and 3), while it is ~10 for a pure 5th-order dispersion. We found that the dispersion limit where the simple field retrieval method based on Eqs. (4–6) starts to fail can be characterized by an inverse power law relationship between the spectral bandwidth and the amount of GDD (nonlinear spectral phase) with the exponent being the dispersion order. This is shown in detail for different spectral shapes in the Supplementary Information (Section S1).

Further constraints are required to use the field retrieval algorithm for pulses with larger GDD or time-bandwidth products by including cross-correlation components at different harmonic orders. This generalized multi-order extension of the retrieval algorithm can increase the practical dispersion limit by several times at the cost of amplifying noise and systematic errors, since it uses cross-correlation components between higher powers of the electric field. A detailed discussion of the multi-order retrieval algorithm and its performance can be found in the Supplementary Information (Sections S2 and S3).

### Retrieval robustness in the presence of noise

In order to assess the susceptibility of the PENGUIN method to noise under realistic experimental conditions, we apply additive and multiplicative noises to a noiseless IAC trace calculated in the time domain via Eq. (1) using a single noise parameter *σ* for simplicity:

7 $$\tilde I_{{{{\mathrm{IAC}}}}}^\sigma \left( \tau \right) = I_{{{{\mathrm{IAC}}}}}\left( \tau \right)\left[ {1 + I_ \times ^\sigma \left( \tau \right)} \right] + I_{{{{\mathrm{IAC}}}}}^{{{{\mathrm{max}}}}}\left( 0 \right)I_ + ^\sigma \left( \tau \right)$$

where $I_{{{{\mathrm{IAC}}}}}^{{{{\mathrm{max}}}}}\left( 0 \right) = {\int}_{ - \infty }^{ + \infty } {\left| {2E\left( t \right)} \right|^{2n}} dt$ is the peak value of the balanced version of the IAC signal (*s* = 1) at time-zero according to Eq. (1). $I_ + ^\sigma (\tau )$ and $I_ \times ^\sigma (\tau )$ denote statistically independent Gaussian white noise signals centered around zero for the additive and multiplicative parts, respectively, with a standard deviation of *σ*. The additive noise is scaled by the peak of the balanced version of the IAC signal (*s* = 1) in order to model the loss of signal-to-noise ratio due to the required attenuation of one of the pulses (*s* < 1) for the PENGUIN field retrieval. Therefore, the additive noise dominates for low-contrast IAC signals using small balance factors *s*, while the additive and multiplicative noises have equal amplitudes in the *s* = 1 limit.

**Fig. 4:**
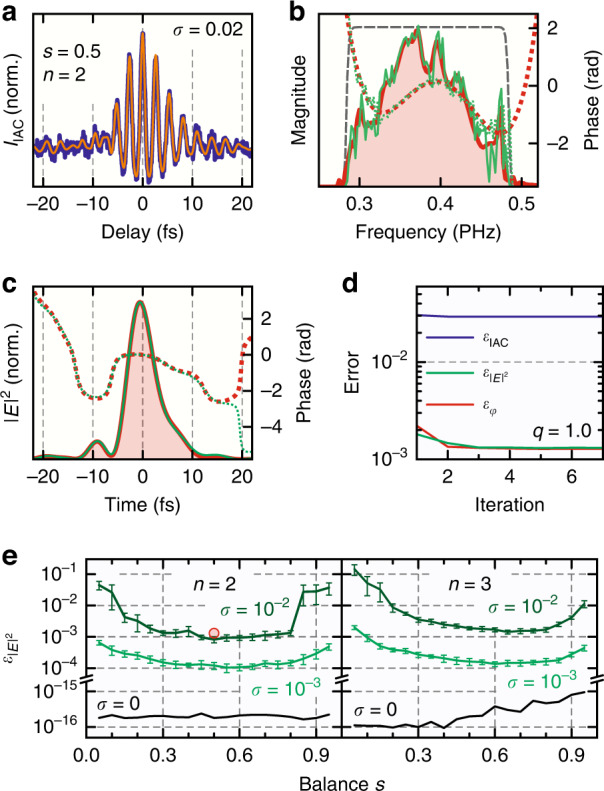
Retrieval of a few-cycle low-dispersion pulse for *n* = 2 and *s* = 0.5 in the presence of noise. **a** Reference (dark blue) and retrieved (orange) IAC traces in the time domain for a strong noise background (*σ* = 0.02) according to Eq. (7). **b** Reference (red) and retrieved (green) field spectra and the fundamental component filter used (dashed gray line). Magnitudes are shown as solid lines and phases as dotted lines. **c** Reference (red) and retrieved (green) temporal profiles of the electric field intensity and phase. The linear component of the temporal phase ($\omega _0t$) has been removed. **d** Error quantities as defined in Eqs. (10–12). The phase error is shown in radians, while the other errors are dimensionless (but are all shown on the same scale). **e** Field error as a function of the balance factor *s* for different noise values shown for *n* = 2 (left) and *n* = 3 (right). Error bars denote standard deviations. The case corresponding to the example shown in a-d is marked by a red circle

As an example, the retrieval of the same low-dispersion pulse that was presented in Fig. 3 is shown in Fig. 4a–d in the presence of strong noise (*σ* = 0.02) for *n* = 2 and *s* = 0.5. The IAC with applied noise according to Eq. (7) is depicted in Fig. 4a (dark blue) in the time domain together with the retrieved IAC trace (orange) showing an excellent match. Note that high-frequency noise components are removed in the retrieved trace by the spectral filter used in the retrieval (dashed gray line in Fig. 4b). Figure 4b, c show the reference (red) and retrieved (green) electric fields in the frequency and time domains, respectively. The short-period noise imparted onto the retrieved spectrum results in low-amplitude, large-period modulation of the time-domain signal, mostly outside the main few-cycle pulse, thus the retrieval quality within the pulse duration is excellent. The retrieval converges within only a few iterations (see Fig. 4d) because of the noise background, and the error values (see Materials and Methods) are only slightly improved after the initial field guess. This is because the initial guess, which is the fundamental component of the IAC itself, already matches the retrieved field reasonably well given the noise magnitude. Furthermore, the noise background is uniformly distributed over the entire 400-fs simulation range, giving it a significant weight in the error calculation compared to the few-cycle pulse duration.

The balance factor *s* has a crucial impact on the retrieval quality in the presence of noise since it directly affects the signal-to-noise ratio available in the experiment. A small balance factor maximizes the spectral phase contrast contained in the IAC by suppressing all cross-correlation components other than $I_{1,0}$ and a constant background; in the strong-field limit (high nonlinear order *n*) this eliminates the need for field retrieval^16^. However, this comes at the cost of a reduced nonlinear signal at the detector (by a factor of up to 2^2*n*^ in the *s* → 0 limit) as well as a reduced contrast of the $I_{1,0}$ signal with respect to the constant background, making the $I_{1,0}$ component more susceptible to intensity fluctuations of the background signal. On the other hand, even though a large balance factor near unity provides the strongest nonlinear signal and highest signal contrast, the phase contrast is reduced with increasing *s* and vanishes completely for a perfectly balanced IAC (*s* = 1). Therefore, there must exist an optimum value for the balance factor *s*, which is shown in Fig. 4e for *n* = 2 and *n* = 3 at different noise levels (the result from the example in Fig. 4a–d is marked with a red circle). The retrieval for each data point was repeated 10 times with different random noise signals to obtain the statistical spread; only the intensity error is shown, since the phase error exhibits comparable behavior. Notably, the retrieval error for the noiseless case is independent of *s* for *n* = 2 since the signal and phase contrast are essentially infinite in this case (within the numerical accuracy). The noiseless error for *n* = 3 increases slightly with *s*, since higher-order cross-correlation components have to be removed from the fundamental IAC peak to obtain $I_{1,0}$ (see Materials and Methods), which introduces some error with increasing *s*, as the $I_{1,0}$ signal becomes buried by the higher-order components. For finite noise values of *σ* = 10^−3^ and *σ* = 10^−2^, covering typical experimental conditions, the retrieval error clearly shows a minimum around *s* = 0.6 for *n* = 2 and *s* = 0.7 for *n* = 3, implying that about 40–50% of the optical power can be preserved in the attenuated pulse for optimum retrieval. The usable range of balance factors for which the error is not more than twice its minimum value is roughly between *s* = 0.3 and *s* = 0.8 in all cases. The same range is found for highly dispersed pulses that require multi-order retrieval. The weak dependence of the retrieval quality on the balance factor greatly relaxes the requirement to fine-tune it for a particular experiment; and the possibility to use relatively large balance factors, retaining most of the IAC signal, makes the PENGUIN method suitable for a wide variety of ultrafast optical field metrology applications. A detailed analysis of the retrieval performance in the presence of noise for highly dispersed pulses can be found in the Supplementary Information (Section S3).

### Experimental demonstration

In order to experimentally demonstrate and validate the new PENGUIN technique, we performed a series of unbalanced-intensity IAC measurements with a varying balance factor *s* using ~2.5-nJ few-cycle Ti:sapphire oscillator pulses and a 10 µm thin β-BaB_2_O_4_ (BBO) crystal as a nonlinear medium for type-I SHG (*n* = 2). The laser pulses were dispersion-minimized via chirped mirrors and a wedge pair, leaving only uncompensated nonlinear chirp. IAC scans were obtained with an actively phase-stabilized Mach–Zehnder interferometer capable of ~35-as root-mean-square stability of the optical delay over a long scan range^47^; the balance factor was set by adjusting a variable ND filter in one of the arms. The two delayed pulses were then collinearly focused into the BBO crystal, and the SHG radiation was separated from the fundamental via a short-pass filter. We chose a 400-fs delay range, which provides a reasonably high spectral resolution after Fourier transformation (2.5 THz), as well as a 0.2-fs step size for sub-cycle temporal resolution. By using a spectrometer as a detector, the same experimental setup is capable of recording an interferometric FROG (IFROG) spectrogram according to Eq. (13) (see Materials and Methods) when the two interferometer arms are adjusted to the same power (*s* = 1); this serves as a validation for the PENGUIN field retrieval method (*s* < 1). To ensure identical experimental conditions for the two different retrieval techniques, we used the same spectrometer as a detector for all measurements. Integrating the measured spectrograms over frequency is equivalent to using a spectrally integrating detector, which yields IAC traces suitable for the PENGUIN retrieval algorithm.

For a proper comparison, both retrieval methods use an identical nonlinear efficiency calibration of the measurement system (see Materials and Methods). Here, we applied a method commonly used in FROG to obtain the nonlinear efficiency curve by comparing the frequency marginal (i.e., the integral over delay time) of a conventional (non-collinear) FROG spectrogram to the autoconvolution of the independently measured fundamental spectral intensity^52^. The conventional FROG spectrogram is contained within the collinear IFROG measurement and can be extracted by Fourier filtering and subtracting the SHG spectrum^36,37^. The resulting nonlinear efficiency curve is essentially flat because of the large phase-matching bandwidth of the thin (10 µm) BBO crystal with a slow roll-off above 800 THz (below 375 nm), and thus has little impact on the field retrieval accuracy. Both methods employ “blind” retrieval, i.e., using only the measured spectrogram or IAC traces, respectively, without any external constraints on the retrieved fundamental field or its spectrum.

**Fig. 5:**
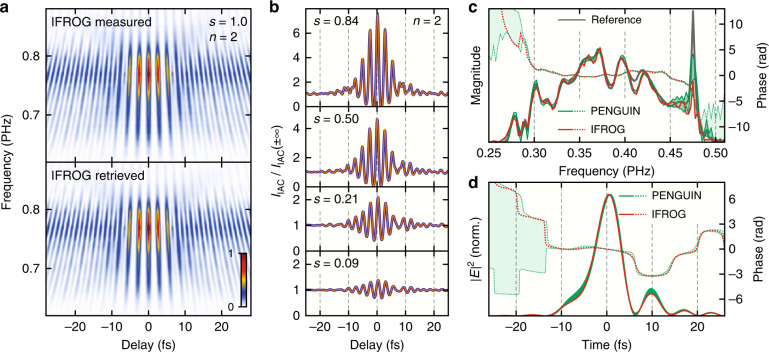
Experimental demonstration of field retrieval using the PENGUIN technique in comparison with the IFROG method for *n* = 2. **a** Measured (top) and retrieved (bottom) balanced IFROG spectrograms (*s* = 1). **b** Representative measured (dark blue) and retrieved (orange) IAC traces for various balance factors *s* using the PENGUIN technique. **c** Retrieved fields in the frequency domain for the IFROG measurement (red) and a series of PENGUIN measurements (green). Magnitudes are shown as solid lines and phases as dotted lines. The green bands bound nine separate measurements with balance factors between *s* = 0.21 and *s* = 0.84 (see text). The independently measured fundamental spectral magnitude is shown in gray for reference. **d** Time-domain representation of the retrieved fields in c showing the intensity envelopes (solid lines) and temporal phases (dotted lines) with the linear component (*ω*_0_*t*) removed. The FWHM duration of the intensity envelope is around 6.4 fs for both retrieval methods

Field retrieval from the IFROG measurement (*s* = 1) was performed with a ptychographic retrieval algorithm originally developed for conventional FROG^53^, which we adapted for IFROG^47^. It provides fast convergence and accurate results while being suitable for an unmodified IFROG spectrogram without the need to separate or filter its harmonic components^36,37,39^. Figure 5a shows an excellent agreement between the measured (top) and retrieved (bottom) IFROG spectrograms; the retrieved field is depicted in red in Fig. 5c, d in the frequency and time domains, respectively.

Figure 5b shows IAC measurements for different balance factors *s* < 1 (dark blue) normalized to the DC background signal, $I_{{{{\mathrm{IAC}}}}}\left( { \pm \infty } \right)$, after integrating the respective spectrograms over frequency. The field was retrieved according to the algorithm described in Eqs. (4–6) but with the nonlinear efficiency curve applied (see Materials and Methods), and the retrieved IAC traces (orange) show an excellent match with the measurements. In addition, the retrieval algorithm automatically recovers the balance factor *s* and the linear spectral phase of the IAC (i.e., the CEP difference between the two pulses and the global time offset) by minimizing the IAC error after each iteration. The CEP difference between the two pulses can also be recovered from the balanced IFROG measurement because of its interferometric nature. However, the direction of time can only be revealed for *s* < 1 where a non-trivial spectral phase is preserved. The fields retrieved with the PENGUIN method for nine different measurements with *s* = 0.21, 0.29, 0.41, 0.50, 0.56, 0.62, 0.67, 0.75, and 0.84 are very close to each other and are therefore represented as green bands bounding all nine measurements in Fig. 5c, d in the frequency and time domains, respectively. Balance values outside this range result in a worse retrieval quality due to reduced signal contrast (low *s*) or phase contrast (high *s*). All individual measurements are shown in detail in the Supplementary Information (Section S5). The small spread of the retrieved field over a large range of balance factors confirms our simulation results in Fig. 4e and allows retaining most of the optical power and signal contrast in the experiment (e.g., for *n* = 2 and *s* = 0.84, the combined two-pulse power is 85% and the signal contrast is 96% compared to *s* = 1).

All retrieved fields using the PENGUIN method (green) very closely match the retrieved field using IFROG (red), which serves as an independent validation of our novel technique. The nine PENGUIN measurements within the green bands yield a mean full-width-at-half-maximum (FWHM) duration of the temporal intensity envelope of 6.45 ± 0.16 fs (the error denotes the standard deviation within the nine measurements), which is almost identical to the 6.44 fs FWHM duration retrieved from the IFROG measurement (solid lines in Fig. 5d). In addition, both retrieval methods are in good agreement with the separately measured spectral magnitude of the laser field (gray line in Fig. 5c). Small systematic deviations due to imperfect spectrometer calibration (e.g., around 415 THz) affect both retrieval methods in the same way, and the PENGUIN technique appears to be more sensitive to narrow spectral features, such as the peak around 475 THz.

## Discussion and conclusions

We presented a novel optical field characterization technique based on an unbalanced-intensity nonlinear IAC, which preserves spectral phase information and enables complete field retrieval with a rapid iterative algorithm. Simulations show a wide usable range of balance factors, which is confirmed by experiments, as well as a high robustness to noise for low-dispersion pulses. We validated this new technique experimentally, demonstrating that the retrieved electric field of few-cycle broadband laser pulses is in excellent agreement with the well-established FROG method.

The PENGUIN technique facilitates a substantial simplification of scientific instruments for the characterization of ultrafast optical fields, specifically the elimination of a spectrometer as the main detector. A spectrally integrating detector can also provide higher sensitivity and dynamic range for weak signals compared to spectrally resolved devices. Almost any existing nonlinear IAC setup is easy to modify for PENGUIN measurements, either by inserting a ND filter in one of the arms or by using beam splitters with different splitting ratios, effectively converting it into a complete optical field characterization device for most practical ultrashort laser pulses similar to FROG or SPIDER but with significantly less complexity and cost. The rapid field retrieval algorithm allows live pulse shape monitoring when a fast optical delay scan is employed, and the delay accuracy can be maintained with a co-propagating continuous-wave reference laser^47^ if necessary. Furthermore, single-shot IAC acquisition with no moving parts is readily available for Fourier-transform spectroscopy applications using a Wollaston prism to map the autocorrelation delay onto the transverse position on a linear photodetector array^54^. PENGUIN can be implemented with this technique simply by changing the polarization of the input beam with respect to the Wollaston prism and placing a nonlinear medium at the line focus. A nonlinear photodetector (e.g., a large-bandgap photodiode or line detector) can be used instead of a separate nonlinear medium to further simplify the setup.

More importantly, the PENGUIN technique opens the door for new and exciting scientific applications of ultrafast optical field metrology where the required nonlinear interaction is inaccessible by spectroscopy, e.g., mapping out ultrafast optical fields via non-radiative processes such as plasmon-enhanced nonlinear photoemission from metallic nanostructures^45–49^. In that regard, our new approach provides a potential alternative to sub-cycle sampling with attosecond pulses^50,51^.

## Materials and methods

### Extraction of the fundamental cross-correlation component, ***I***_**1**,**0**_, from the IAC

The $I_{1,0}$ component can be directly recovered from the IAC signal for *n* = 2 and *s* < 1 by spectral filtering around the fundamental peak of the IAC and complex scaling, since in that case it only contains the sum of $I_{1,0}$ and its complex conjugate scaled by *s*^2^ (i.e., $I_{1,1} = s^2\overline {I_{1,0}}$):

8 $$I_{1,0}\left( \omega \right) = F\left( \omega \right)\left[ {\frac{{{{{\mathrm{Re}}}}\left[ {I_{{{{\mathrm{IAC}}}}}\left( \omega \right)} \right]}}{{1 + s^2}} + i\frac{{{{{\mathrm{Im}}}}\left[ {I_{{{{\mathrm{IAC}}}}}\left( \omega \right)} \right]}}{{1 - s^2}}} \right]$$

with *F*(*ω*) being a suitable filter window to isolate the fundamental peak around $\omega _0$ (e.g., dashed gray line in Fig. 3a). For higher nonlinear orders *n*, the fundamental IAC peak contains additional higher-order cross-correlation components, i.e., $I_{1,k > 0}$ in Eq. (2), which are calculated during the field retrieval using the current field guess and subtracted from the IAC signal filtered around the fundamental peak in order to iteratively recover the $I_{1,0}$ signal. Since all higher-order fundamental cross-correlation components are scaled by some power of *s* with respect to the $I_{1,0}$ signal, the error from approximating these components quickly tends towards zero as Eq. (6) converges towards the original fundamental field.

### Retrieval error quantities

In order to quantify the retrieval error, we use normalized mean absolute error values in the time domain. For the IAC error, we define a normalized background-free form of the IAC signal:

9 $$\hat I_{{{{\mathrm{IAC}}}}}\left( \tau \right) = \frac{{I_{{{{\mathrm{IAC}}}}}\left( \tau \right) - \left( {s^{2n} + 1} \right)\mathop {\int }\nolimits_{ - \infty }^{ + \infty } \left| {E^n\left( t \right)} \right|^2dt}}{{\left( {\left( {s + 1} \right)^{2n} - s^{2n} - 1} \right)\mathop {\int }\nolimits_{ - \infty }^{ + \infty } \left| {E^n\left( t \right)} \right|^2dt}}$$

where the delay-independent IAC background, $I_{{{{\mathrm{IAC}}}}}\left( { \pm \infty } \right) = \left( {s^{2n} + 1} \right)\mathop {\int }\nolimits_{ - \infty }^{ + \infty } \left| {E^n\left( t \right)} \right|^2dt$, and the contrast ratio, $I_{{{{\mathrm{IAC}}}}}\left( 0 \right)/I_{{{{\mathrm{IAC}}}}}\left( { \pm \infty } \right) = \left( {s + 1} \right)^{2n}/\left( {s^{2n} + 1} \right)$, were used, which follow from Eq. (1). The normalized form in Eq. (9) is always unity at *τ* = 0 and approaches zero for *τ* → ±∞, allowing quantitative comparison independent of *n* and *s*. The IAC error is then given by

10 $$\varepsilon _{{{{\mathrm{IAC}}}}} = \frac{1}{T}{\int \nolimits_{ - \frac{T}{2}}^{\frac{T}{2}}} \left| {\hat I_{{{{\mathrm{IAC}}}},{{{\mathrm{ret}}}}.}\left( \tau \right) - \hat I_{{{{\mathrm{IAC}}}},{{{\mathrm{ref}}}}.}\left( \tau \right)} \right|d\tau$$

with $\hat I_{{{{\mathrm{IAC}}}},{{{\mathrm{ret}}}}.}$ and $\hat I_{{{{\mathrm{IAC}}}},{{{\mathrm{ref}}}}.}$ being the retrieved and reference normalized IACs, respectively, and *T* the relevant temporal extent of the measurement or simulation. Similarly, for a normalized complex electric field of the form $\hat E\left( t \right) = \left[ {\left| {E\left( t \right)} \right|/E_0} \right]{{{\mathrm{exp}}}}\left[ {i\omega _0t + i\varphi \left( t \right)} \right]$ with a peak amplitude of $E_0$ and temporal phase $\varphi \left( t \right) = \arg \left[ {E\left( t \right)} \right] - \omega _0t$, we define the intensity and phase errors of the field as

11 $$\varepsilon _{\left| E \right|^2} = \frac{1}{T}{\int \nolimits_{ - \frac{T}{2}}^{\frac{T}{2}}} \left| {\left| {\hat E_{{{{\mathrm{ret}}}}.}\left( t \right)} \right|^2 - \left| {\hat E_{{{{\mathrm{ref}}}}.}\left( t \right)} \right|^2} \right|dt$$

12 $$\varepsilon _\varphi = \frac{1}{T}{\int \nolimits_{ - \frac{T}{2}}^{\frac{T}{2}}} \left| {\varphi _{{{{\mathrm{ret}}}}.}\left( t \right) - \varphi _{{{{\mathrm{ref}}}}.}\left( t \right)} \right|\left| {\hat E_{{{{\mathrm{ref}}}}.}\left( t \right)} \right|^2dt$$

Note that the phase error, $\varepsilon _\varphi$, is weighted by the intensity profile of the reference field in order to make it comparable to the other error quantities, which are inherently weighted by the temporal distribution of the respective signals.

The IAC signal is invariant with respect to the linear spectral phase of the electric field, that is, its CEP and time offset. Therefore, we perform a simple linear fit to adjust these parameters of the retrieved field to match the linear component of the spectral phase of the reference field after each iteration in order to assess the retrieval quality in the simulations.

### Nonlinear spectral efficiency correction

All characterization methods for broadband optical pulses are usually affected by a non-uniform spectral efficiency of the nonlinear process and detector leading to systematic errors in the measurement. Therefore, the nonlinear efficiency curve of the measurement system has to be determined and accounted for in the field retrieval. Since the spectral efficiency is an intrinsic property of the particular experiment, it can either be calibrated externally prior to the measurement or determined directly from the measurement by providing the separately measured fundamental spectral intensity as a constraint. The latter approach is typically used with spectrally resolved field characterization methods such as FROG and d-scan^41,52,55^ and is also available for the spectrally integrating PENGUIN technique; this is discussed in detail in the Supplementary Information (Section S4).

Unlike a spectrogram, the spectrally integrated IAC signal cannot be corrected for the nonlinear efficiency of the measurement system before retrieving the electric field. However, a known nonlinear efficiency curve can be applied to the calculated cross-correlation components and gating field used in the retrieval process enabling correct field retrieval from an IAC signal affected by a non-uniform spectral efficiency. This generally requires computing the spectrally resolved form of Eq. (1):

13 $$I_{{{{\mathrm{IAC}}}}}\left( {\omega ^\prime ,\tau } \right) = \left| {{\int \nolimits_{ - \infty }^{ + \infty }} \left( {sE\left( t \right) + E\left( {t - \tau } \right)} \right)^ne^{ - i\omega ^\prime t}dt} \right|^2$$

Here, $\omega ^\prime$ denotes the frequency domain after a Fourier transform with respect to *t* at each delay step *τ*. For *s* = 1, Eq. (13) describes the IFROG spectrogram^36,37^ (see Fig. 5a). The binomial expansion of Eq. (13) contains spectrogram components corresponding to their spectrally integrated counterparts in Eq. (1), all of which can be easily scaled by a nonlinear efficiency filter, $C_n\left( {\omega ^\prime } \right)$. Integrating Eq. (13) multiplied by $C_n\left( {\omega ^\prime } \right)$ over $\omega ^\prime$ yields the one-dimensional IAC (or any of its cross-correlation components) in the time domain as in Eq. (1) but with the nonlinear efficiency curve applied. Note that the calculation of the full spectrogram increases the computational effort by about a factor of *N* (*N* being the number of IAC delay steps) compared to the field retrieval using only one-dimensional quantities.

For field retrieval according to Eqs. (4–6) from the $I_{1,0}$ component of an IAC that is affected by a nonlinear efficiency curve, the gating field must be calculated with the same nonlinear efficiency curve applied. This can be done without computing a two-dimensional spectrogram, because the gating field, $E_{{{{\mathrm{G}}}}(1,0)}\left( t \right) = E\left( t \right)\left| {E^{n - 1}\left( t \right)} \right|^2 = E^n\left( t \right)\overline {E^{n - 1}\left( t \right)}$, contains the *n*th-order harmonic field. Applying the nonlinear efficiency curve to $E^n\left( t \right)$ via Fourier transform yields a filtered gating field, $\tilde E_{{{{\mathrm{G}}}}(1,0)}\left( t \right) = {{{\mathcal{F}}}}^{ - 1}\left\{ {{{{\mathcal{F}}}}\left\{ {E^n\left( t \right)} \right\}C_n\left( {\omega ^\prime } \right)} \right\}\overline {E^{n - 1}\left( t \right)}$, to be used in Eq. (6) for retrieval. For *n* = 2, the $I_{1,0}$ component is directly extracted from the IAC via Eq. (8); thus, field retrieval can be accomplished with the filtered gating field alone and does not require computing spectrograms. For higher nonlinear orders *n*, the $I_{1,0}$ component has to be obtained iteratively during the retrieval by subtracting higher-order cross-correlation components from the IAC at the fundamental frequency. Applying the nonlinear efficiency curve to these components with *k* > 0 requires calculating their spectrograms using Eq. (13) which are then multiplied with $C_n\left( {\omega ^\prime } \right)$ and integrated over frequency.

## Supplementary information

Supplementary Information

## Data Availability

A reference implementation of the PENGUIN code for the simulation of unbalanced-intensity IAC traces and field retrieval is available upon request from the authors.
