# Supplementary material for: Complete characterization of ultrafast optical fields by phase-preserving nonlinear autocorrelation
Source: Light Sci Appl. 2022 Sep 20;11:277. doi: 10.1038/s41377-022-00978-3 (PMC9485149; doi:10.1038/s41377-022-00978-3)
Supplement: Supplementary file 1 — Supplementary Information [file 41377_2022_978_MOESM1_ESM.pdf]

# Supplementary Information for Complete characterization of ultrafast optical fields by phase-preserving nonlinear autocorrelation

**Alexander Gliserin[1,2,4*], Soo Hoon Chew[1,2,4], Seungchul Kim[1,2*], Dong Eon Kim[3,4*]**

1) *Department of Optics and Mechatronics Engineering, College of Nanoscience and Nanotechnology, Pusan National University, 2 Busandaehak-ro 63beon-gil, Busan 46241, South Korea*
2) *Department of Cogno Mechatronics Engineering, College of Nanoscience and Nanotechnology, Pusan National University, 2 Busandaehak-ro 63beon-gil, Busan 46241, South Korea*
3) *Department of Physics, Center for Attosecond Science and Technology, Pohang University of Science and Technology, 77 Cheongam-ro, Pohang, 37673, South Korea*
4) *Max Planck Center for Attosecond Science, Max Planck POSTECH/Korea Research Initiative, 77 Cheongam-ro, Pohang, 37673, South Korea*

* E-mails: alex@mpk.or.kr, s.kim@pusan.ac.kr, kimd@postech.ac.kr

## S1. Dispersion limitations of the single-order field retrieval algorithm

The rapid and accurate single-order field retrieval method based on Eqs. (4)-(6) in the main text using only the $I_{1,0}$ component of the IAC is limited to pulses with a gating field that is spectrally broader than the fundamental field. This is true for pulses near the Fourier-transform limit or generally if most of the energy is confined within a spectral region of relatively low GDD regardless of the time-bandwidth product. Since the gating field is some power of the fundamental field and therefore is always shorter in the time domain, it can only become narrower in the frequency domain if the fundamental field is significantly dispersed, i.e., if its spectral phase has a large nonlinear component over an extended part of the spectral energy distribution. In this case the single-order retrieval algorithm fails to retrieve the true electric field and instead converges onto a different, less dispersed, solution for the electric field which satisfies Eq. (3) in the main text. Thus, at least two electric field solutions exist in this regime. The simulation shown in Fig. S1a illustrates the break-down of the single-order retrieval method above a "critical" amount of dispersion for $n = 2$ and $s = 0.5$ using a Gaussian spectral magnitude at 400 THz central frequency with 80 THz FWHM intensity bandwidth and a purely linear chirp (quadratic spectral phase), i.e., a constant GDD (red lines in Fig. S1a, right column). A bifurcation point emerges around 19 fs$^2$ of GDD, above which a wrong lower-dispersion solution is retrieved (note the flatter spectral phase of the retrieved field; dotted green lines), even though the retrieval algorithm converges for all GDD values. This wrong

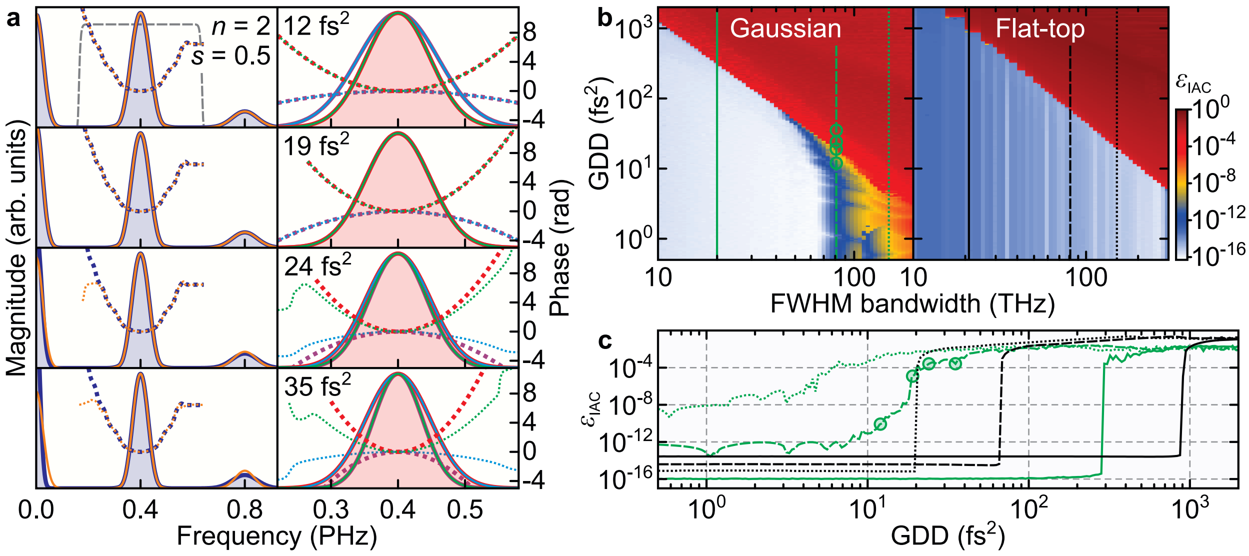


**Fig. S1** Limitations of single-order field retrieval for high-dispersion pulses. **a** Reference (dark blue) and retrieved (orange) IAC spectra for $n$ = 2 and $s$ = 0.5 (left panels) using a Gaussian spectrum at 400 THz central frequency with 80 THz FWHM intensity bandwidth and different amounts of GDD (purely linear chirp or quadratic spectral phase); reference (red) and retrieved (green) fundamental fields and corresponding reference (purple) and retrieved (blue) gating fields (right panels). The fundamental filter used for retrieval is depicted as a dashed gray line. Magnitudes are shown as solid lines and phases as dotted lines; flat phase segments are not shown. **b** IAC error as defined in Eq. (10) in the main text as a function of GDD and FWHM bandwidth for a Gaussian spectrum (left) and a near-rectangular flat-top spectrum (right), respectively. A sharp boundary for the correct field retrieval is evident at the critical GDD value, which scales with the bandwidth following an inverse power law. **c** Lineouts from b showing the IAC error as a function of GDD for bandwidth values around 20 THz (solid), 80 THz (dashed), and 150 THz (dotted) for the Gaussian spectrum (green) and flat-top spectrum (black), respectively, denoted by the corresponding vertical lines in b. The IAC errors for the four example panels in a are marked by green circles in b and c.

solution reproduces the fundamental-frequency peak of the original IAC but fails to reproduce its other harmonic peaks and is therefore trivial to detect by comparing all harmonic peaks of the retrieved IAC (orange; left column in Fig. S1a) with the reference IAC (dark blue). Note that at the critical GDD value the spectral bandwidth of the gating field (purple/blue) approaches that of the fundamental field (red/green). In the special case of a Gaussian spectrum and linear chirp, even the shape of their spectral magnitudes becomes identical (see the 19 fs$^2$ example). Above the critical GDD the reference gating field (purple) becomes spectrally narrower than the fundamental field (red), while the retrieved gating field (blue) cannot become spectrally narrower than the fundamental field, even for large GDD values, resulting in a retrieved field (green) with a narrower spectral bandwidth and flatter spectral phase than the reference field. Notably, in this regime there is a match in spectral bandwidth between the reference fundamental field (red) and retrieved gating field (blue) as well as between the retrieved fundamental field (green) and reference gating field (purple). It is

yet unclear whether additional electric field solutions exist for Eq. (3) in the main text above the critical dispersion apart from trivial ambiguities, such as the linear spectral phase of the field.

Figure S1b demonstrates the transition from the correct solution to the wrong solution for a Gaussian spectrum (left) and a flat-top spectrum (right), respectively, after the convergence of the single-order field retrieval, in which the IAC error (see Eq. (10) in the main text) is shown as a function of both GDD and spectral bandwidth. The flat-top spectrum used here is essentially a rectangular spectrum with slightly rounded corners to ensure a smooth spectral shape. All spectra have a central frequency of 400 THz. We show the IAC error rather than the field intensity error (Eq. (11) in the main text) because all error quantities behave almost identically in the absence of noise (see Fig. 3c in the main text) and only the IAC error is accessible in a real-world measurement as a gauge for a successful retrieval. The critical GDD is evident as a sharp edge separating regions of successful retrieval from failed retrieval (i.e., yielding the wrong solution) by an IAC error jump of many orders of magnitude. The retrieval quality for the Gaussian spectrum slightly deteriorates for large spectral bandwidths even below the critical GDD, since the large bandwidth leads to a significant overlap between adjacent harmonics in the IAC spectrum, which is not the case for the flat-top spectrum having a steep roll-off outside its spectral bandwidth. Still the IAC error jumps by several orders of magnitude at the critical GDD even for large-bandwidth Gaussian spectra but exhibits a somewhat broadened transition region. Remarkably, the critical GDD clearly shows an inverse power law dependence on the spectral bandwidth with an exponent of 2 for both spectral shapes, which appears to be fundamentally linked to the nonlinear order of the spectral phase (quadratic in the case of constant GDD or linear chirp). Applying, e.g., a purely cubic spectral phase (i.e., third-order dispersion) leads to an inverse power law dependence with an exponent of 3. The scaling factor of this relationship between GDD and spectral bandwidth (intercept of the critical GDD edge in the double logarithmic plot in Fig. S1b) depends on the spectral shape and is highest in the flat-top case, i.e., the same bandwidth yields a larger critical GDD value, since this shape has the strongest confinement of the spectral power distribution for a given bandwidth.

Figure S1c shows lineouts taken from Fig. S1b for both Gaussian (green) and flat-top (black) spectral shapes for FWHM intensity bandwidth values around 20 THz (solid), 80 THz (dashed), and 150 THz (dotted) as denoted by the corresponding vertical lines in Fig. S1b. The IAC error values for the examples in Fig. S1a (80 THz FWHM bandwidth Gaussian spectrum with different GDD values) are shown as green circles in Figs. S1b and c.

## S2. Multi-order field retrieval

The field solution to Eq. (3) in the main text, which uses only the $I_{1,0}$ component of the IAC, bifurcates above a spectrum-dependent critical dispersion, yielding the wrong field for highly dispersed pulses with the single-order field retrieval algorithm (see section S1). Thus, further constraints are required to force retrieval of the correct solution in this regime. Here, we expand the single-order field retrieval method (based on Eqs. (4)-(6) in the main text) by including other harmonic components of the IAC as constraints, thereby extending the practicality of the PENGUIN field retrieval technique to moderately dispersed pulses well beyond the critical dispersion.

In principle, every cross-correlation component, $I_{m,k}$ (see Eq. (2) in the main text), can be isolated from the IAC signal during the field retrieval by spectral filtering around the respective harmonic and subtracting all other components at that harmonic, which are approximated using the current field guess at each iteration. Similarly to Eq. (5) in the main text, the generalized gating field for any cross-correlation component, $I_{m,k}$, is obtained from the current field guess, $E^{\langle i\rangle}(t)$:

$$E_{\mathrm{G}(m,k)}^{\langle i\rangle}(t) = \left[E^{\langle i\rangle}(t)\right]^m \left|\left[E^{\langle i\rangle}(t)\right]^{n-k-m}\right|^2 \tag{S1}$$

Then, analogously to the fundamental field retrieval in Eq. (6) in the main text, the field power, $E_{m,k}(t)$, contained within this cross-correlation component can be iteratively decorrelated:

$$E_{m,k}^{\langle i+1\rangle}(t) = \left[E^m(t)|E^k(t)|^2\right]^{\langle i+1\rangle} = \mathcal{F}^{-1}\left\{\frac{I_{m,k}(\omega)}{\binom{n}{k}\binom{n}{k+m} s^{2k+m}\overline{\mathcal{F}\left\{E_{\mathrm{G}(m,k)}^{\langle i\rangle}(t)\right\}}}\right\} \tag{S2}$$

Note that the field power, $E_{m,k}(t)$, has a magnitude of $|E(t)|^{2k+m}$ and a temporal phase given by $m\cdot\arg[E(t)]$. Therefore, taking the $(2k+m)^{\mathrm{th}}$ root of the magnitude of $E_{m,k}(t)$ yields the magnitude of the fundamental field, and dividing the unwrapped phase of $E_{m,k}(t)$ by $m$ (except for $m = 0$) yields the temporal phase of the fundamental field. Finally, the fundamental field magnitudes and phases retrieved from each cross-correlation component are combined via a weighted average with weighting factors $w_{m,k}$ to obtain the fundamental field guess for the next iteration:

$$E^{\langle i+1\rangle}(t) = \left[\frac{q}{w_{\mathrm{mag}}}\sum_{m=0}^{n}\sum_{k=0}^{n-m} w_{m,k}\left|E_{m,k}^{\langle i+1\rangle}(t)\right|^{\frac{1}{2k+m}}\right]\exp\left[\frac{i}{w_\varphi}\sum_{m=1}^{n-1}\sum_{k=0}^{n-m}\frac{w_{m,k}}{m}\arg\left[E_{m,k}^{\langle i+1\rangle}(t)\right]\right]$$

$$+E^{\langle i\rangle}(t)(1-q), \qquad w_{\mathrm{mag}} = \sum_{m=0}^{n}\sum_{k=0}^{n-m} w_{m,k}\,,\; w_\varphi = \sum_{m=1}^{n-1}\sum_{k=0}^{n-m} w_{m,k} \tag{S3}$$

with $q$ being a parameter controlling the rate of convergence as introduced in Eq. (6) in the main text. Note that the phase contributions are omitted for $m = 0$ and $m = n$ in Eq. (S3) because the $I_{0,k}$ components only retrieve powers of the field magnitude squared ($E_{0,k}(t) = |E^k(t)|^2$) and the $I_{n,0}$ component is a magnitude-squared quantity and thus provides no spectral phase information (see Eq. (2) in the main text). Furthermore, all components with $k > (n-m)/2$ are complex-conjugate replica of lower-order components and therefore can be ignored (weights set to zero). This generalized *multi-order* PENGUIN retrieval algorithm only converges if the retrieved fundamental electric field satisfies all cross-correlation components at different harmonics used in Eq. (S3), that is, when all retrieved field powers, $E_{m,k}(t)$, yield the same fundamental field (or its magnitude). The simple single-order ($I_{1,0}$) retrieval method according to Eqs. (4)-(6) in the main text is contained in the generalized version described in Eqs. (S1)-(S3) by setting all weights to zero except $w_{1,0}$.

Using higher field powers contained in the different IAC harmonics to constrain the field solution has some practical limitations, since it requires taking roots to obtain the fundamental field (or its magnitude), which greatly amplifies noise and errors from spectrally filtering and isolating the cross-correlation components. In practice, using a single additional harmonic component at the lowest possible order, $I_{0,1}$, which retrieves $|E(t)|^2$, is sufficient for $n > 2$, since in that case the DC peak of the IAC ($m = 0$) contains a non-trivial spectral phase. For $n = 2$, both the $I_{0,1}$ and the $I_{2,0}$ components have to be used, since neither contains spectral phase information (see, e.g., Fig. 2c in the main text) and is therefore by itself insufficient to retrieve $|E(t)|^2$, but can force the correct field solution when all three unique IAC components (including the fundamental, $I_{1,0}$) are combined.

Figure S2 demonstrates this for $n = 2$ (a-d) and $n = 3$ (e-h), comparing the single-order retrieval with the multi-order method (top and bottom panels, respectively) using $s = 0.5$ in all cases. The electric field used here has the same spectral magnitude as in Fig. 3 in the main text (taken from a measurement), but with a significant amount of constant GDD (i.e., a parabolic spectral phase) of 40 fs$^2$ over the entire spectrum causing a near-ideal linear temporal chirp of the pulse (the temporal phase is not exactly parabolic because the spectrum is non-Gaussian). Using only the fundamental IAC peak (spectral filters shown by dashed gray lines in Figs. S2a and e), the single-order retrieval converges onto the wrong solution for the electric field (Figs. S2a-c, e-g, top panels) as explained in section S1. This solution satisfies Eq. (3) in the main text and therefore perfectly matches the fundamental peak of the reference IAC but fails to reproduce all other harmonic peaks (see also Fig. S1a) and significantly underestimates the duration of the original pulse (Figs. S2c and g, top

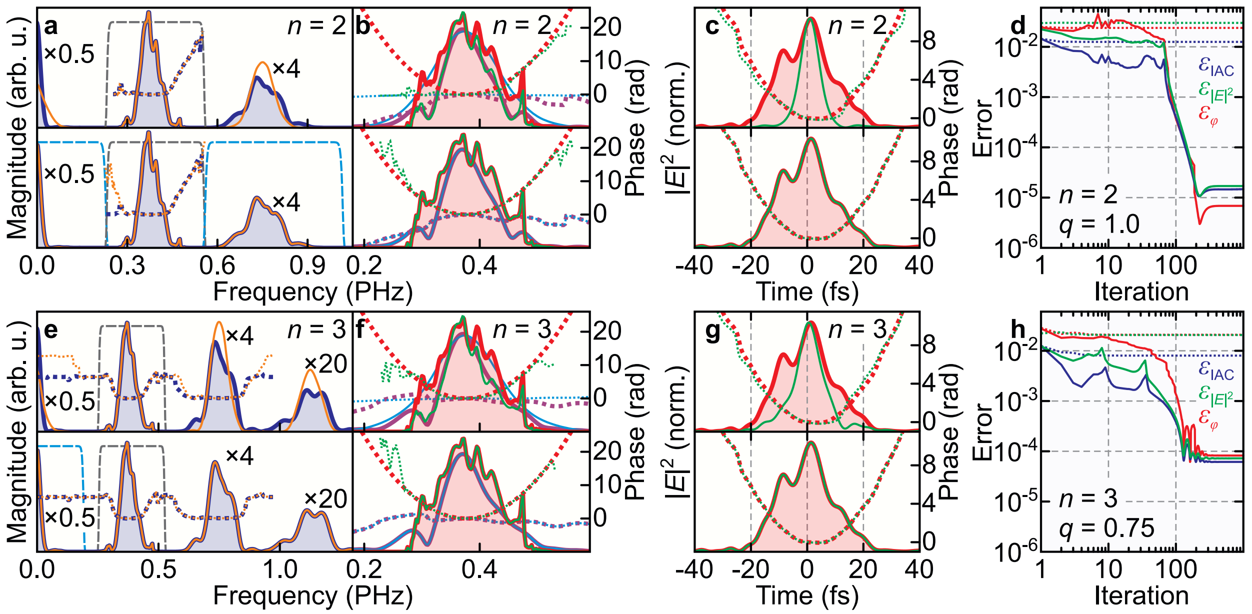


**Fig. S2** Comparison between single-order and multi-order field retrieval for $s$ = 0.5 and a significantly dispersed pulse (parabolic spectral phase with 40 $fs^2$ of constant GDD). **a, e** Reference (dark blue) and retrieved (orange) IAC spectra as well as spectral filters for the fundamental (dashed gray lines) and additional (dashed blue lines) harmonic components. All harmonic peaks except the fundamental are scaled by different factors for clarity. Magnitudes are shown as solid lines and phases as dotted lines; flat phase segments are not shown. **b, f** Reference (red) and retrieved (green) electric fields and the respective reference (purple) and retrieved (blue) gating fields. **c, g** Reference (red) and retrieved (green) temporal profiles of the electric field intensity (solid lines) and phase (dotted lines) with the linear component ($\omega_0 t$) removed. The top panels in a-c and e-g show the single-order field retrieval, while the bottom panels show the multi-order retrieval. **d, h** Error quantities as defined in Eqs. (10)-(12) in the main text for the single-order (dotted lines) and multi-order (solid lines) retrieval. The phase error is shown in radians, while the other errors are dimensionless (but are all shown on the same scale).

panels) while roughly matching the temporal phase. Although the single-order retrieval converges, the error quantities as defined in Eqs. (10)-(12) in the main text (Figs. S2d and h, dotted lines) are essentially not reduced since the wrong field solution is retrieved. In contrast, when using additional IAC harmonics in Eq. (S3) (spectral filters shown by dashed blue lines in Figs. S2a and e), the true fundamental electric field solution is retrieved, whose IAC matches the reference IAC spectrum at all harmonic peaks (Figs. S2a-c, e-g, bottom panels). The filter widths are chosen to minimize the IAC error and the optimum relative weights are $w_{0,1}$ = 200 and $w_{2,0}$ = 100 for $n$ = 2 and $w_{0,1}$ = 500 for $n$ = 3, respectively, with $w_{1,0}$ = 1 in both cases. This means that the additional harmonic components mainly retrieve the temporal magnitude of the field, while the fundamental IAC peak provides its phase. All error quantities are now reduced by 2-3 orders of magnitude after the multi-order retrieval (Figs. S2d and h, solid lines), which is well below practical experimental noise levels. Numerically near-perfect retrieval as in the low-dispersion single-order case cannot be achieved

here due to the error introduced by taking roots of the field powers retrieved from the additional harmonic peaks. Furthermore, the retrieval converges slower and somewhat non-monotonously, since no feedback from the IAC error is used in the algorithm, thus convergence has to be judged based on the change of the IAC error over some number of iterations.

## S3. Multi-order retrieval performance in the presence of noise

Next, we investigate the field retrieval performance for pulses with different amounts of dispersion and noise using a fixed balance factor of $s = 0.5$, which is well within the optimum range according to Fig. 4e in the main text, and compare the nonlinear orders of $n = 2$ and $n = 3$. To this end, a pulse with the same spectral magnitude as in Fig. 3 in the main text was used but with varying amounts of constant GDD (i.e., linear chirp) and applied noise. Here, Eq. (7) in the main text was slightly modified such that the additive noise part ($I_{+}^{\sigma}$) was now scaled by the time-zero peak signal of the unbalanced-intensity IAC, $I_{\mathrm{IAC}}(0) = \int_{-\infty}^{+\infty} |(s + 1)E(t)|^{2n} dt$, instead of the balanced IAC ($s = 1$), which results in the same contribution of the additive and multiplicative noise components and therefore allows a quantitative comparison between the $n = 2$ and the $n = 3$ case for the same noise parameter $\sigma$. Single-order ($I_{1,0}$) field retrieval was used for low dispersion and multi-order retrieval as introduced in section S2 was used for high dispersion beyond the bifurcation point of the single-order solution, which in this case is around critical GDD values of 14 fs$^2$ and 16 fs$^2$ for $n = 2$ and $n = 3$, respectively (see Fig. S3b). As in the case of the balance scan in Fig. 4e in the main text, the retrieval was repeated 10 times for each parameter pair (GDD and noise strength) with randomly seeded noise signals to obtain the statistical spread. Outliers were removed if their retrieved field error exceeded twice the median value for this parameter pair, amounting to a total of about 3% of removed samples. For strong noise and high dispersion, the retrieval may not converge, leading to oscillatory or even chaotic behavior of the error values. Furthermore, the ideal harmonic weights and spectral filter widths generally change with dispersion and noise and need to be optimized for each case. Our retrieval strategy in this regime employed a numerical optimization algorithm based on differential evolution to automatically adjust these parameters during the retrieval with the objective of minimizing the IAC error. Meanwhile the field guess yielding the lowest IAC error regardless of the iteration step was held in memory and returned as the retrieval result if a better field guess with a lower IAC error could not be found within a certain number of iterations (several thousands in our case). This approach served solely to automate the retrieval

process over a large parameter space and in practice adjusting the filter and weight parameters for a particular experiment can be easily done manually using the same stopping criteria.

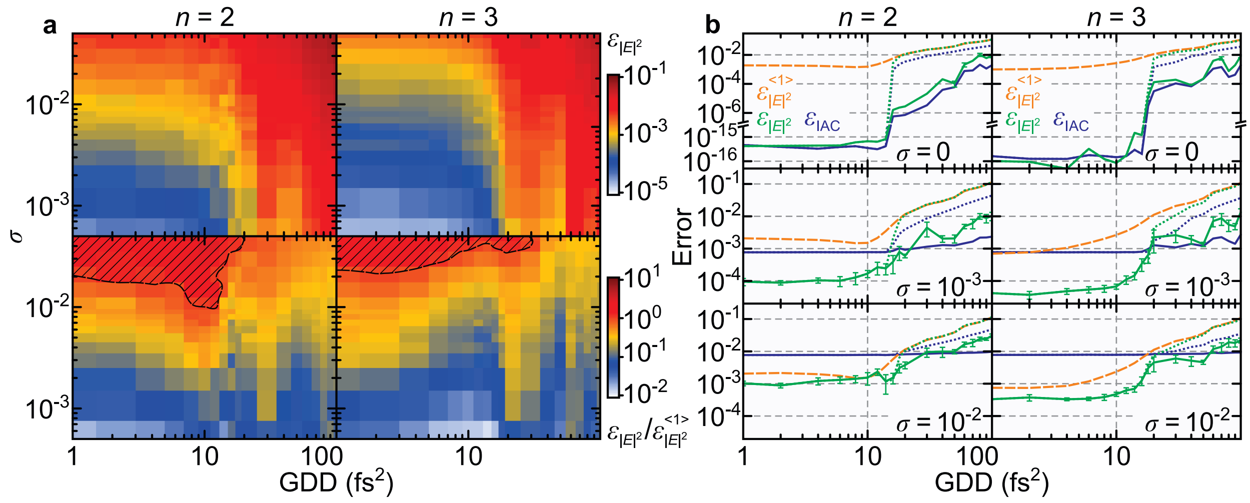


**Fig. S3** Field retrieval quality for different dispersion and noise values for $n$ = 2 and $n$ = 3 using a balance factor of $s$ = 0.5 in all cases. **a** Top panels: logarithmic two-dimensional map of the retrieved field error (see Eq. (11) in the main text) as a function of GDD and the noise parameter $\sigma$. Bottom panels: retrieved field error normalized to the first-guess field error showing the relative improvement by the retrieval. The contour of the striped area denotes a normalized field error of 1. **b** Lineouts at different noise values, including $\sigma$ = 0, depicting the retrieved IAC error (dark blue) and field error (green) according to Eqs. (10) and (11) in the main text as well as the first-guess field error (dashed orange lines). The dotted lines show error values for single-order ($I_{1,0}$) retrieval, while the solid lines show multi-order retrieval above the critical GDD. Error bars denote standard deviations.

Figure S3a (top panels) shows the resulting two-dimensional map of the field error, $\varepsilon_{|E|^2}$ (see Eq. (11) in the main text), for up to 100 fs$^2$ of GDD and up to a noise value of $\sigma$ = 0.05. The error generally increases with GDD as expected even for single-order retrieval below the critical GDD value because the field error is inherently weighted by the pulse duration. The single-order and multi-order retrieval regimes are clearly separated by a jump in the field error at the critical GDD value (similar to Fig. S1b), comparable to adding about an order of magnitude of noise to the single-order retrieval. Here, the field error is consistently lower for $n$ = 3 compared to $n$ = 2 below the critical GDD while it becomes comparable above the critical GDD. Lineouts of the field error map are shown in Fig. S3b (green lines) for different noise levels including the noiseless case. Without noise, the transition at the critical GDD value is accompanied by a steep jump of all error values by many orders of magnitude, since multi-order retrieval adds some systematic error even if the IAC is noise-free. Here, the $n$ = 2 case shows anomalous behavior by yielding smaller error values than for $n$ = 3 immediately above the critical GDD value. An additional first-guess field error, $\varepsilon^{\langle 1\rangle}_{|E|^2}$, is

shown by the dashed orange lines, which is given by Eq. (11) in the main text evaluated after the initial field guess before any retrieval. The subsequent retrieval is considered a failure if the retrieved field error cannot be reduced below the first-guess field error. For comparison, the error quantities for single-order retrieval above the critical GDD (see Fig. S1c and top panels in Fig. S2) are shown as dotted lines, and the single-order field error quickly approaches the first-guess error above the critical GDD for all noise levels, since it converges onto the wrong field solution. Using multi-order retrieval, the field error is improved by up to four orders of magnitude for $n = 2$ and up to two orders of magnitude for $n = 3$ above the critical GDD in the noiseless case. In the presence of noise, the transition at the critical GDD value is clearly present but not as steep and becomes less pronounced with increasing noise. Notably, the IAC error, $\varepsilon_{\mathrm{IAC}}$ (dark blue lines), is almost independent of GDD in the presence of noise even above the critical GDD, which is partly because the IAC signal experiences only little temporal broadening due to GDD (unlike the electric field). It also signifies that the multi-order retrieval algorithm optimizes the match of the retrieved IAC to the reference IAC regardless of the match between the different field powers, $E_{m,k}(t)$, retrieved from the different IAC components. Thus, the algorithm can potentially be improved by forcing a match between the field powers as an additional constraint. Likewise, the first-guess field error, $\varepsilon_{|E|^2}^{\langle 1 \rangle}$, is almost independent of the noise level for temporally well-localized IAC signals with respect to the simulation range. The initial field guess is directly derived from the IAC signal and therefore closely follows its temporal shape but with most of the high-frequency noise removed due to spectral filtering around the fundamental peak. This robustness to noise of the initial field guess prevents our retrieval method from improving the first-guess field error for low GDD at the highest noise levels, which is shown in the bottom panels of Fig. S3a, where the retrieved field error has been normalized to the first-guess field error. The striped areas denote over-unity values of the normalized field error, i.e., where the retrieval fails to improve the initial field guess or even increases the error after the initial guess. Here, the noise-resistant initial field guess provides already a reasonable match with the fundamental field given the noise level, while the retrieval quality is substantially worsened by forcing it to match the spectral noise pattern of the IAC. This advantage of the initial guess vanishes for highly dispersed pulses, since the first guess significantly underestimates the pulse duration in that case, while the multi-order retrieval method recovers the pulse duration relatively well, even if the exact pulse shape is poorly matched in the presence of strong noise. Thus, the multi-order retrieval improves the first-guess field error over almost the entire parameter space above the critical GDD.

## S4. Self-calibration of the nonlinear spectral efficiency

Any optical pulse characterization method must account or correct for a non-uniform spectral efficiency of the measurement system due to a limited bandwidth of the nonlinear process and spectral response of the detector, especially when measuring broadband ultrashort pulses. Both quantities are usually difficult to obtain, either by modeling or independent calibration. Spectrally resolved field characterization methods such as FROG and d-scan allow not only easy nonlinear efficiency correction of the spectrogram but also the determination of an unknown nonlinear efficiency curve directly from the measurement using the separately provided fundamental spectral intensity (or magnitude) as a constraint [1–3]. A similar self-calibration of the nonlinear spectral efficiency is possible with the PENGUIN method if the fundamental spectral magnitude is known by exploiting the $n^{\text{th}}$-order harmonic signal contained in the IAC.

Both the single-order (Eqs. (4)-(6) in the main text) and multi-order (Eqs. (S1)-(S3)) field retrieval algorithms can be used to retrieve the nonlinear efficiency curve, $C_n(\omega)$, for a particular measured IAC signal when provided with an independently measured spectral magnitude of the fundamental field. The retrieval starts as a regular field retrieval with a flat nonlinear efficiency curve, $C_n^{\langle 1\rangle}(\omega) = 1$. After each ($i^{\text{th}}$) iteration the spectral magnitude of the retrieved field guess, $E^{\langle i+1\rangle}(t)$, is replaced with the externally provided spectral magnitude of the fundamental field, $S(\omega)$:

$$E_S^{\langle i+1\rangle}(t) = \mathcal{F}^{-1}\{S(\omega)\exp\left(i\arg\left[\mathcal{F}\{E^{\langle i+1\rangle}(t)\}\right]\right)\} \tag{S4}$$

Then the $n^{\text{th}}$-order harmonic spectral intensity of this spectrally constrained field is calculated and compared to the $n^{\text{th}}$-order harmonic peak contained in the measured IAC spectrum (i.e., the Fourier transform of the IAC signal according to Eq. (2) in the main text). The ratio between them yields an approximation of the nonlinear efficiency curve to be used for the next iteration:

$$C_n^{\langle i+1\rangle}(\omega) = \frac{I_{\text{IAC}}(\omega)F_n(\omega)}{\left|\mathcal{F}\left\{\left[E_S^{\langle i+1\rangle}(t)\right]^n\right\}\right|^2} \tag{S5}$$

with $F_n(\omega)$ being a suitable filter window to isolate the $n^{\text{th}}$-order harmonic peak of the IAC (see the dashed turquoise lines in Figs. S4b and d). Each iteration thus provides both a new field guess, $E^{\langle i+1\rangle}(t)$, as well as a new guess of the nonlinear efficiency curve, $C_n^{\langle i+1\rangle}(\omega)$, which are both used in the subsequent retrieval (see Materials and Methods in the main text). It is evident that if the

retrieval converges onto the correct field solution, Eq. (S5) yields the true nonlinear efficiency curve, $C_n(\omega)$, since the $n^{\text{th}}$-order harmonic peak of the measured IAC spectrum is simply the $n^{\text{th}}$-order harmonic spectral intensity of the fundamental field (see Eq. (2) in the main text) scaled by the nonlinear efficiency curve. Providing the independently measured fundamental spectral magnitude, $S(\omega)$, is required to disambiguate the relationship between $E^{\langle i \rangle}(t)$ and $C_n^{\langle i \rangle}(\omega)$ during the retrieval, which would otherwise have to solve the Fourier relation in Eq. (6) in the main text (or analogously in Eq. (S2)) for two unknowns. Using the $n^{\text{th}}$-order harmonic spectral intensity contained within the IAC spectrum as a calibration reference implies that a large balance factor $s$ is desirable to preserve a high signal contrast of the $n^{\text{th}}$-order harmonic peak for accurate self-calibration. Alternatively, a separately measured $n^{\text{th}}$-order harmonic spectrum can also be used as a reference as long as it is affected by the same spectral efficiency as the IAC measurement.

Figure S4 demonstrates the retrieval of the nonlinear efficiency curve by using the fundamental spectral magnitude as a constraint. Here, the same simulated electric field was used as in Fig. 3 in the main text, i.e., a typical spectral magnitude of a broadband few-cycle laser pulse (taken from a measurement) with a synthetic low-dispersion polynomial spectral phase suitable for single-order field retrieval (Eqs. (4)-(6) in the main text). In order to demonstrate the retrieval of the nonlinear efficiency curve, we applied a Gaussian nonlinear efficiency filter (dashed dark gray lines) with 200 THz FWHM bandwidth centered around 780 THz and 1.2 PHz for $n = 2$ and $n = 3$, respectively, when calculating the reference IAC (dark blue) for $s = 0.5$ as laid out in the Materials and Methods section in the main text. The nonlinear efficiency filter was deliberately offset from the centers of the respective $n^{\text{th}}$-order harmonic peaks in order to emphasize the effect of a non-uniform spectral response of the measurement system, and the bandwidth was chosen to simulate a significantly spectrally constricted nonlinear conversion process. The field retrieval results without correcting for the nonlinear efficiency (i.e., using a flat nonlinear efficiency curve and no spectral constraint) are shown in Figs. S4a and c. The retrieval converges and yields a rough match between the reference (dark blue) and retrieved (orange) IACs as well as the reference (red) and retrieved (green) fundamental fields. The lack of a nonlinear efficiency correction is most apparent when comparing the reference (purple) and retrieved (blue) $n^{\text{th}}$-order harmonic spectral intensities, since the reference one (extracted from the IAC) is affected by the nonlinear efficiency curve.

Figures S4b and d depict the retrieval of the nonlinear efficiency curve (bright green) at different iteration steps of the retrieval algorithm. Here, the reference fundamental spectral

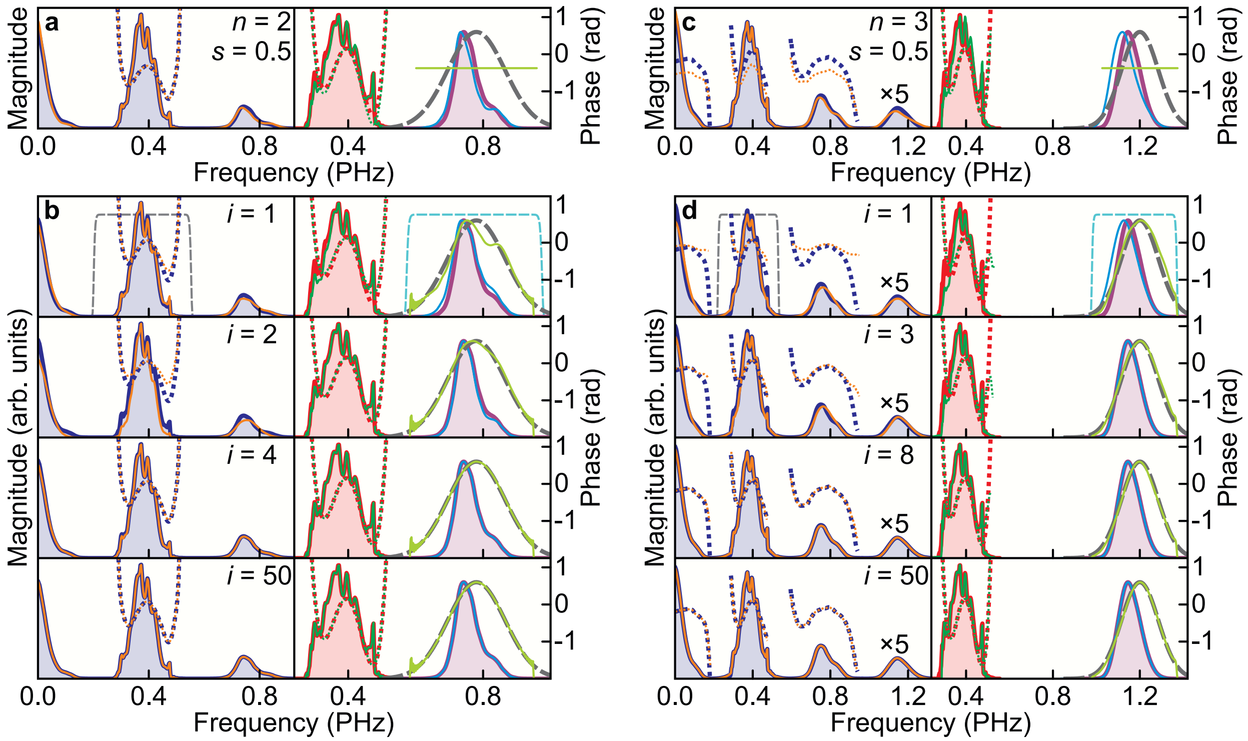


**Fig. S4** Field retrieval from IAC traces affected by Gaussian-shaped nonlinear efficiency curves (dashed dark gray lines) for $s = 0.5$ and $n = 2$ (a, b) as well as $n = 3$ (c, d). Each panel pair shows reference (dark blue) and retrieved (orange) IAC spectra (left panels), reference (red) and retrieved (green) fundamental fields as well as reference (purple) and retrieved (blue) spectral intensities of the second-order (a, b) and third-order (c, d) harmonic fields, respectively, scaled by the corresponding nonlinear efficiency curve (right panels). Magnitudes (intensities) are shown as solid lines and phases as dotted lines; flat phase segments are not shown. The third-order harmonic IAC peak is scaled by a factor of 5 for clarity. **a, c** Fully converged field retrieval result using a flat nonlinear efficiency curve (bright green). **b, d** Simultaneous retrieval of the field and the nonlinear efficiency curve (bright green) using the reference spectral magnitude (red) as a constraint shown after different iteration steps *i*. The spectral filters used for the fundamental field retrieval and for the nonlinear efficiency retrieval are shown as dashed gray and turquoise lines, respectively.

magnitude (red) was used as a constraint to obtain the nonlinear efficiency curve by comparing the retrieved $n^{\text{th}}$-order harmonic spectral intensity of the (constrained) field with the $n^{\text{th}}$-order harmonic peak contained within the reference IAC spectrum (purple) according to Eqs. (S4) and (S5). The blue lines show the retrieved $n^{\text{th}}$-order harmonic spectral intensity scaled by the retrieved nonlinear efficiency curve at each iteration step for better comparison with the reference from the IAC. Appropriate filter windows (dashed turquoise lines) were used to isolate the $n^{\text{th}}$-order harmonic peaks from the reference IAC spectra and to constrain the spectral support of the retrieved nonlinear efficiency curve to a region of sufficient signal contrast of the $n^{\text{th}}$-order harmonic spectral intensity. Therefore, the retrieved nonlinear efficiency curve is only well-defined within the bandwidth of the $n^{\text{th}}$-order harmonic spectral intensity. Since this is also the spectral bandwidth of the IAC signal

itself at the detector (before being spectrally integrated), it is sufficient for complete field retrieval as evidenced by a rapid convergence and an excellent match with the reference for both the retrieved fundamental fields as well as the retrieved nonlinear efficiency curves for $n = 2$ and $n = 3$.

Note that any field retrieval method that uses the independently measured spectral magnitude of the fundamental field as a constraint only needs to retrieve the spectral phase for a full optical field characterization. In fact, several widely used techniques, such as d-scan and SPIDER [3–6], only retrieve the spectral phase and thus require the knowledge of the spectral magnitude of the fundamental field by other means. Here, we used the externally provided spectral magnitude solely for the retrieval of the nonlinear efficiency calibration according to Eqs. (S4) and (S5) rather than to explicitly constrain the spectral magnitude of the retrieved field to the external reference. It is trivial to apply this constraint to the retrieved field after each iteration via Eq. (S4), thus employing the PENGUIN technique only for phase retrieval if the spectral magnitude of the fundamental field is known. This provides more accurate and robust field retrieval in the presence of noise as well as a built-in nonlinear efficiency calibration.

## S5. Detailed experimental field retrieval results

While Figs. 5c and d in the main text show the field retrieved by the PENGUIN method as bands bounding nine different measurements with balance factors between $s = 0.21$ and $s = 0.84$, the results of all twelve IAC measurements in this series including higher and lower balance factors are shown in Fig. S5 in individual panels (green). The field retrieved from the IFROG ($s = 1$) measurement (red) and the independently measured fundamental spectral magnitude (gray) are also shown for reference (identical in each panel). A close match between the IFROG and each of the PENGUIN measurements confirms the excellent quality of the field retrieval with the novel method for all balance factors except close to the extrema where the measurements suffer from low signal contrast ($s \gtrsim 0$) or low phase contrast ($s \lesssim 1$), respectively. Systematic deviations from the independently measured spectral magnitude are identical for both methods due to imperfect spectrometer calibration. Given the relatively good retrieval quality even for a balance factor as high as $s = 0.94$, only a small intensity imbalance is needed in most practical cases (depending on the accuracy requirement), thus preserving almost all optical power and signal contrast compared to a balanced ($s = 1$) IAC or IFROG measurement.

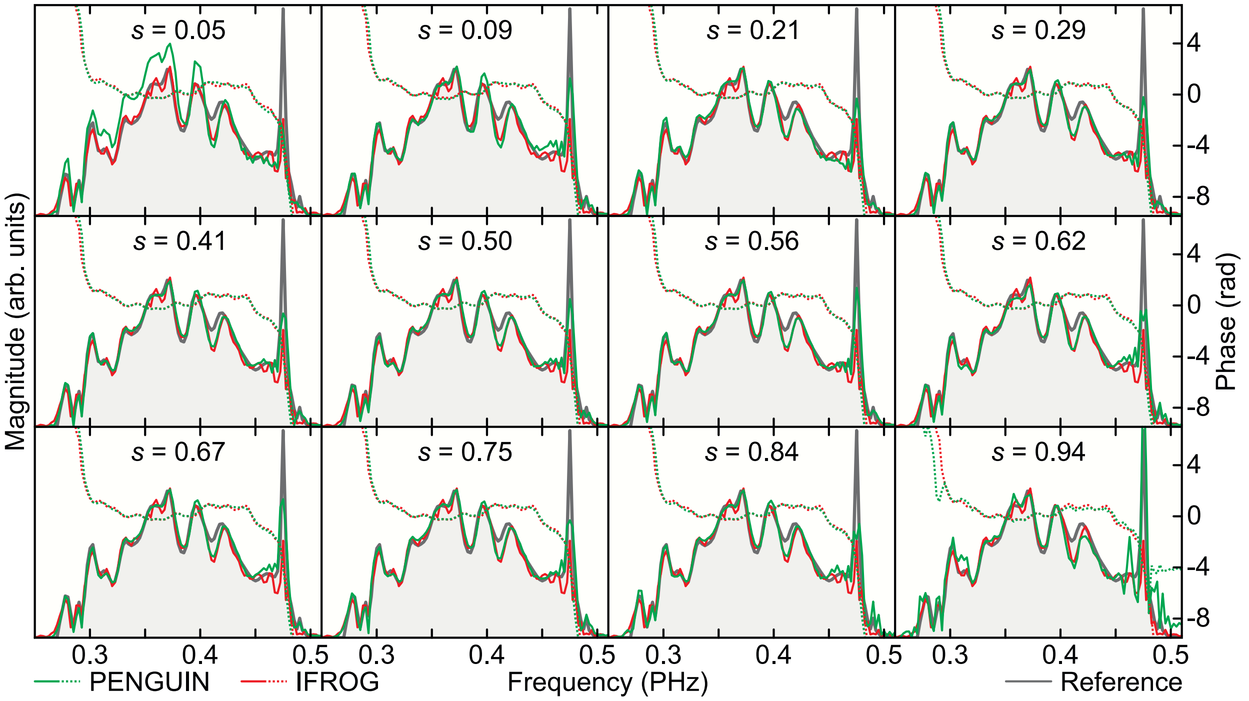


**Fig. S5** Field retrieval results of twelve individual IAC measurements with varying balance factors using the PENGUIN technique in comparison with the IFROG measurement for $n = 2$. Magnitudes are shown as solid lines and phases as dotted lines. The retrieved field using the PENGUIN method (green) is shown for every balance factor together with the IFROG ($s = 1$) measurement (red) as well as the independently measured fundamental spectral magnitude (gray) for reference. The reference measurements (red and gray) are identical in all panels.

## References


[1] K. W. DeLong, R. Trebino, and D. J. Kane, Comparison of ultrashort-pulse frequency-resolved-optical-gating traces for three common beam geometries, *Journal of the Optical Society of America B* **11**, 1595 (1994).

[2] K. W. DeLong, D. N. Fittinghoff, and R. Trebino, Practical issues in ultrashort-laser-pulse measurement using frequency-resolved optical gating, *IEEE Journal of Quantum Electronics* **32**, 1253 (1996).

[3] M. Miranda, T. Fordell, C. Arnold, A. L'Huillier, and H. Crespo, Simultaneous compression and characterization of ultrashort laser pulses using chirped mirrors and glass wedges, *Optics Express* **20**, 688 (2012).

[4] M. Miranda, C. L. Arnold, T. Fordell, F. Silva, B. Alonso, R. Weigand, A. L'Huillier, and H. Crespo, Characterization of broadband few-cycle laser pulses with the d-scan technique, *Optics Express* **20**, 18732 (2012).

[5] C. Iaconis and I. A. Walmsley, Self-referencing spectral interferometry for measuring ultrashort optical pulses, *IEEE Journal of Quantum Electronics* **35**, 501 (1999).

[6] M. E. Anderson, A. Monmayrant, S.-P. Gorza, P. Wasylczyk, and I. A. Walmsley, SPIDER: A decade of measuring ultrashort pulses, *Laser Physics Letters* **5**, 259 (2008).
